# Optimization of the heat recovery performance of enhanced geothermal system based on PSO-GA-BP neural networks and analytic hierarchy process

**DOI:** 10.1038/s41598-025-07509-1

**Published:** 2025-07-01

**Authors:** Ling Zhou, Jingchao Sun, Yanjun Zhang, Yunjuan Chen, Honglei Lei

**Affiliations:** 1 School of Civil Engineering, Shandong Jianzhu University, Jinan, 250101 China; 2 Key Laboratory of Building Structural Retrofitting and Underground Space Engineering (Shandong Jianzhu University), Jinan, 250101 China; 3 College of Construction Engineering, Jilin University, Changchun, 130026 China; 4 Shandong Jianda Engineering Appraisal and Reinforeement Design Co., Ld., Jinan, 250014 China

**Keywords:** Enhanced geothermal system, Optimization, PSO-GA-BPNN models, Analytic hierarchy process, Production performance, Energy science and technology, Engineering

## Abstract

Numerical simulation is the most commonly used method to predict the power generation capacity of EGS during geothermal energy extraction. However, it is time-consuming to optimize the scheme only by comparing the numerical simulation methods, and it is difficult to determine the globally optimal operation strategy. In this study, five key parameters including well spacing, water injection rate, injection temperature, fracture permeability and fracture spacing are considered. Based on the numerical simulation data, optimized Back-Propagation Neural Network (BPNN) prediction models combining the Particle Swarm Optimization (PSO) and the Genetic Algorithm (GA) were developed to investigate the impact of various factors on the heat recovery performance of a three-horizontal-well EGS in the Zhacang geothermal field. On the basis of these PSO-GA-BPNN models, the weights of the evaluation indexes for each geothermal development were calculated by hierarchical analysis method. In this study, an innovative combination of numerical simulation, PSO-GA-BPNN model, and Analytic Hierarchy Process was proposed to establish an EGS comprehensive optimization method, effectively improving the accuracy and computational efficiency of scheme optimization. The results reveal that predicting EGS with PSO-GA-BPNN models has a good prediction accuracy for each performance index. After a comprehensive comparison, the combination of well spacing of 600 m, water injection rate of 27 kg/s, injection temperature of 58 ℃, fracture permeability of 1 × 10^–10^ m^2^ and fracture spacing of 100 m was identified as the optimal power generation scheme. The EGS power plant is expected to have an installed capacity of 6.05–8.17 MW, with a total generating capacity of 3,163.16 GWh and a levelized cost of electricity of $0.033/kWh. The method is very effective in the development and optimal design of geothermal systems and can also provide a reference for other geothermal projects.

## Introduction

Geothermal energy is a clean renewable energy source stored in the earth’s crust, which is characterized by its flexible use and wide distribution, and plays an important role in addressing the problems of energy shortage, atmospheric pollution and climate change^1,2^. Hot Dry Rock (HDR) is the low-permeability rock mass with a burial depth of 3–10 km and a temperature higher than 180°C^3,4^. It is estimated that global HDR resources contain about 30 times as much thermal energy as oil, gas and coal reserves. The total HDR resources in China are 2.52 × 10^25^ J, equivalent to more than 800 trillion tons of standard coal, which can be applied to power generation, heating and industrial production^5^.

Enhanced Geothermal System (EGS) technology is an efficient method for the economic utilization of hot rocks by using hydraulic, thermal, or chemical stimulation to construct high-permeability artificial fractures in low-permeability reservoirs to increase flow conductivity within the reservoir^6^. Since the first EGS project in the United States at Fenton Hill in 1973, other countries have opened EGS research programs^7^. By 2020, a total of 64 EGS site projects have been established in 20 countries, of which 14 have realized power generation^8^. The total installed capacity of the 5 projects currently in operation is 12.2 MW: the Desert Peak project (2002-present) and Geysers project (2009-present) in the USA, Soultz project in France (1987-present), Landau project in Germany (2003-present), and Habanero project in Australia (2009-present)^9–13^. The reserves of HDR resources in China are very considerable, but the first EGS demonstration site has not yet been built, mainly because the existing fracturing technology cannot transform a thermal reservoir that can be used for long-term resource extraction^14^. In 2013, China Geological Survey conducted a nationwide HDR resource potential evaluation, and made a major breakthrough in HDR exploration and exploitation in the Gonghe-Guide Basin of Qinghai Province^15^. The Qinghai Bureau of Environmental Geology Exploration completed two geothermal boreholes in Zhacang geothermal field in Guide Basin. The bottom temperature was 151.34 °C at 3050.68 m depth in borehole ZR1, and the temperature exceeded 100 °C at 100 m due to the influence of the fault; the highest temperature of 214 °C was obtained at a depth of 4602 m in borehole ZR2^16,17^. The Zhacang geothermal field has an area of 8.4 km^2^ and geothermal resources equivalent to about 1.5 billion tons of standard coal, which means that the Zhacang geothermal field has the potential to build a long-term EGS power plant^18^. Therefore, this field is selected as the case study.

During the operation of an EGS, there undergoes a coupled Thermal–Hydraulic-Mechanical(THM) process in the subsurface fractured rock mass. In order to investigate the complex water flow heat transfer processes in fractured rocks, many scholars have conducted laboratory experiments. Ma et al. and Huang et al^19–21^. explored the heat transfer characteristics of water flowing through rough fracture through the seepage and convective heat transfer experiments, and the results demonstrated that the rough fracture surface produces more heat dissipation when fluid flows through the rock surface. Shu et al.^22^ observed that permeability and heat transfer coefficients decrease with increasing pressure at constant temperature and heat transfer coefficients increase with temperature at constant pressure. However, the scale of the reservoir rock mass is extremely large in actual engineering; the mechanical properties of the rock mass have scale and time effects in the laboratory and actual engineering. Laboratory simulation conditions are usually difficult to realize that the simulation environment matches the actual engineering, so the research results are very different from the actual situation. Although the field in situ testing can accurately reflect the actual situation of the project, it requires a large amount of human, financial, and material resources, so it is necessary to rely on numerical simulation to make up for the shortcomings of this study. At present, numerical simulation methods can be used to solve the complex multi-field coupling problems of sites in the geothermal field based on certain site tests.

The construction of EGS requires huge economic investment, so more and more researchers are committed to improving the power generation capacity of EGS. Nanofluids are used as heat carriers, because the increase in the thermal conductivity of the heat carrier can reduce the depth required for drilling, thereby reducing capital investment^23^. Duan et al.^24^ carried out compressionerosion tests and permeability prediction research, and proposed a practical strategy to strengthen the broken rock above the geothermal well, locate the geothermal well stronger compression-erosion effects, and increase the pumping pressure, which can expand the scale of the geothermal well and improve the mining efficiency of the geothermal well. For the conventional EGS with water as the heat transfer carrier, in order to improve its production efficiency, appropriate planning and design can be carried out in advance. Numerical simulation is the most efficient and least costly method to study the heat production process of EGS and has been widely used in geothermal fields around the world in recent years^25–27^. There are many parameters that affect the performance of EGS, including natural properties, completion parameters and operational parameters^28^. The analysis of the influence of each parameter on the performance index of EGS can provide a basis for optimizing the production scheme. Zhang et al^29^. studied the heat production performance of the Zhacang geothermal field by establishing two horizontal wells and analyzed the effects of fracture spacing, injection temperature, and injection rate on EGS power generation. Lei et al^30^. developed a three-vertical well model for sensitivity analysis of EGS generation performance, the results show that for fully stimulated EGS reservoirs, an optimized EGS with an injection rate of 40 kg/s, a well spacing of 500 m, and an injection temperature of 60 °C is feasible. Nadimi et al^31^. discovered that the injection temperature and flow rate are the two most important parameters for influencing the production temperature and heat recovery after analyzing the other factors. Prior research has focused on the analysis of a single factor, but in reality, the geothermal power generation capacity is influenced by a number of factors, and the relationship between these factors and the geothermal power generation capacity is not a straightforward linear relationship. The single objective optimization of geothermal performance may not acquire a balanced and feasible combination of operational parameters^32^. As a result, multiple factors must be studied simultaneously in order to determine the best exploitation strategy.

In order to determine the optimal EGS production scheme, it is necessary to try as various combinations of key parameters as possible, but this process takes a lot of time for simulation calculations, so some scholars have introduced mathematical models to simplify this process. Asai et al^28^. analyzed the effects of well spacing, fracture spacing, well inclination, injection temperature, and injection rate on the EGS power generation capacity using a second-order polynomial function model. The results showed that the heat recovery factor can be optimized by studying the hierarchy of parameters. Samin et al^33^. integrated finite element analysis and genetic algorithm optimisation technique to evaluate the influence of design parameters in order to achieve optimal EGS reservoir design. Zhou et al^34^. constructed BP artificial neural network models to predict the production temperature and electrical power of EGS. The results show that increasing the injection flow rate, lowering the injection temperature, increasing the distance between injection and production wells, and reducing the fracture permeability can improve the heat recovery within a certain range. Yang et al^35^. proposed a one-dimensional convolutional neural network model and used data augmentation techniques to construct a large-scale multi-scale production temperature dataset for production temperature prediction in a three vertical well geothermal system. Previous studies have shown that ANN has excellent prediction ability in the production temperature and electric power of EGS. However, there are many evaluation indexes of EGS performance, including heat production temperature, flow resistance, power generation, and system thermal efficiency, etc.; the importance of different indexes on the power generation performance of EGS is different. It is necessary to comprehensively evaluate the influencing factors and performance indicators to select the optimal program from the whole situation. Hierarchical analysis is a fair and reasonable decision-making method, which can consider all the performance indicators comprehensively to accomplish the target optimization^36^. Currently, there are few studies on system solution optimization using ANN combined with hierarchical analysis, which is very important to improve the capacity of EGS.

The main objective of this study is to exploit the deep high-temperature geothermal resources in Zhacang geothermal field. According to the regional geology and drilling data, the 4000–4600 m stratum is selected as the target thermal reservoir to establish the three horizontal wells EGS numerical model to simulate the power generation potential for 50 years. The PSO-GA-BPNN model is innovatively combined with the numerical simulation method, which greatly shortens the time of simulating the operation process of EGS. The effects of single factors such as water injection speed, water injection temperature, fracture permeability, well spacing and fracture spacing and the interaction of various factors on the performance indexes (production temperature, heat extraction efficiency, flow resistance, electric power and thermal efficiency) of EGS are comprehensively studied^5,37^. In order to optimize the production strategy of Zhacang geothermal field, an EGS comprehensive evaluation model was proposed. The analytic hierarchy process was used to comprehensively score the five performance indicators of production temperature, heat extraction efficiency, flow resistance, electrical power and thermal efficiency, and a quantitative comprehensive evaluation method was established. Finally, the economic and environmental benefits of the optimal solution obtained based on the method proposed in this paper are analyzed. The research results provide an accurate and effective optimization method for the optimal development model of geothermal resources, which is helpful to improve the energy efficiency of geothermal resources. The proposed method can also be effectively applied to other geothermal development models.

## Background of the study site

**Fig. 1:**
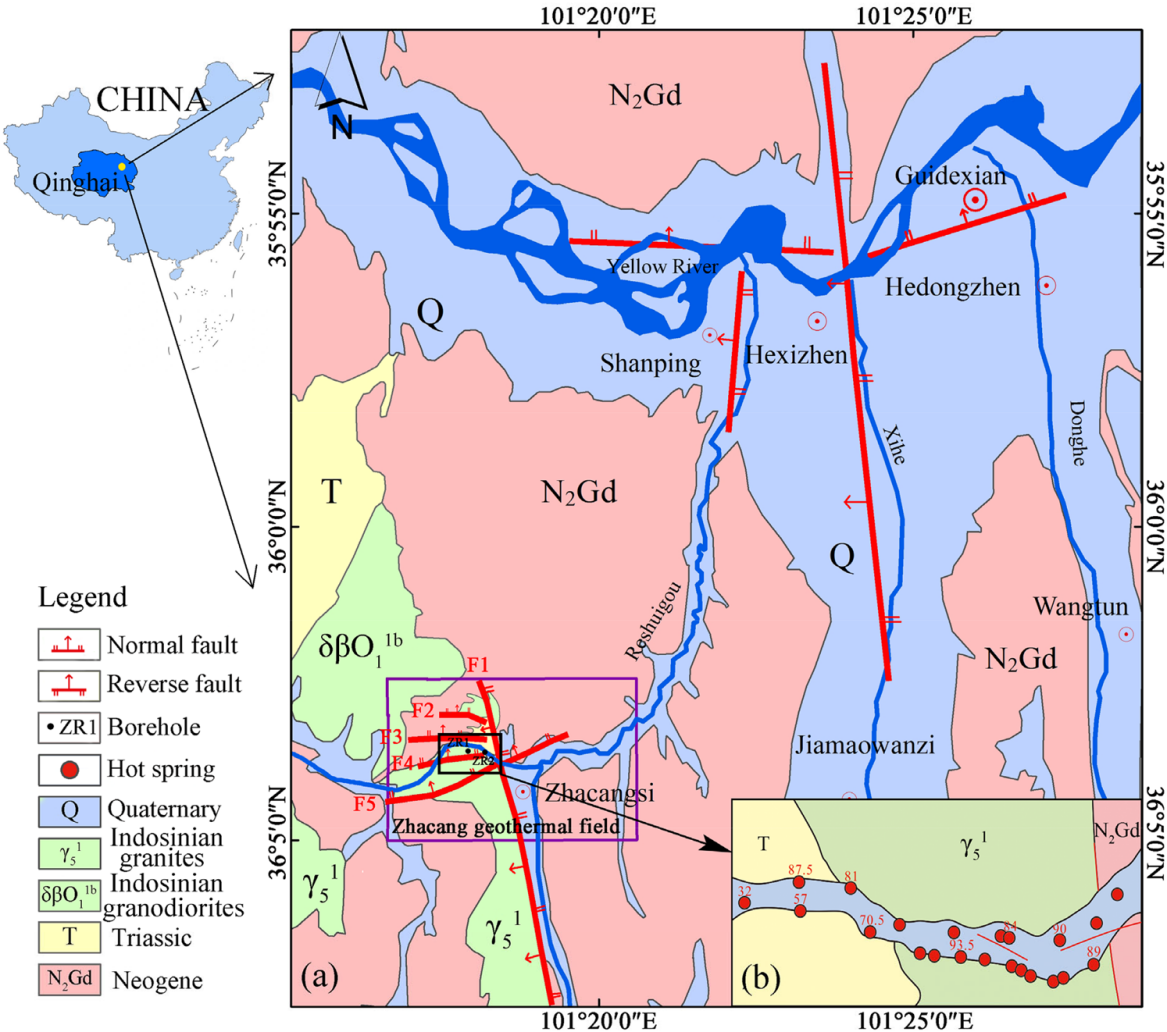
Location of the Guide Basin and Zhacang geothermal field. Maps were drawn by authors, using ArcGIS 10.8 (Environmental Systems Research Institute, USA. https://www.esri.com/).

The Guide Basin in the southeastern part of the Hainan Tibetan Autonomous Prefecture in Qinghai Province is a demonstration area for geothermal development in China. The Zhacang geothermal field is located in the central part of the Guide Basin, about 15 km from the urban area of Guide County (Fig. 1a). Zhacang geothermal field is controlled by the Hexi structural system. The Zhacang fault (F5) is a NE-trending tension-torsional normal fault which is a good channel for the upward transportation of geothermal water. The Reguang fault (F1) is a NNW-trending compression-torsional reverse fault that serves as a natural barrier for geothermal water flow. In the southwestern mountains, atmospheric precipitation seeps into the ground through the near EW-trending fault zones and is heated by a deep heat source, and the water flow is blocked by the Reguang fault and exposed on the surface to form the Zhacang hot springs^38^. There are nearly 30 hot springs distributed within 150 m in the gully (Fig. 1b), with temperatures ranging from 32.0 to 93.5 °C; total flow rates ranging from 8.0 to 15.2 L/s, and containing a variety of trace elements beneficial to the body^39^. The hot springs are well developed and utilized for bathing and irrigation, with sanatoriums constructed due to the springs have good medical function.

### Geothermal characteristics

**Fig. 2:**
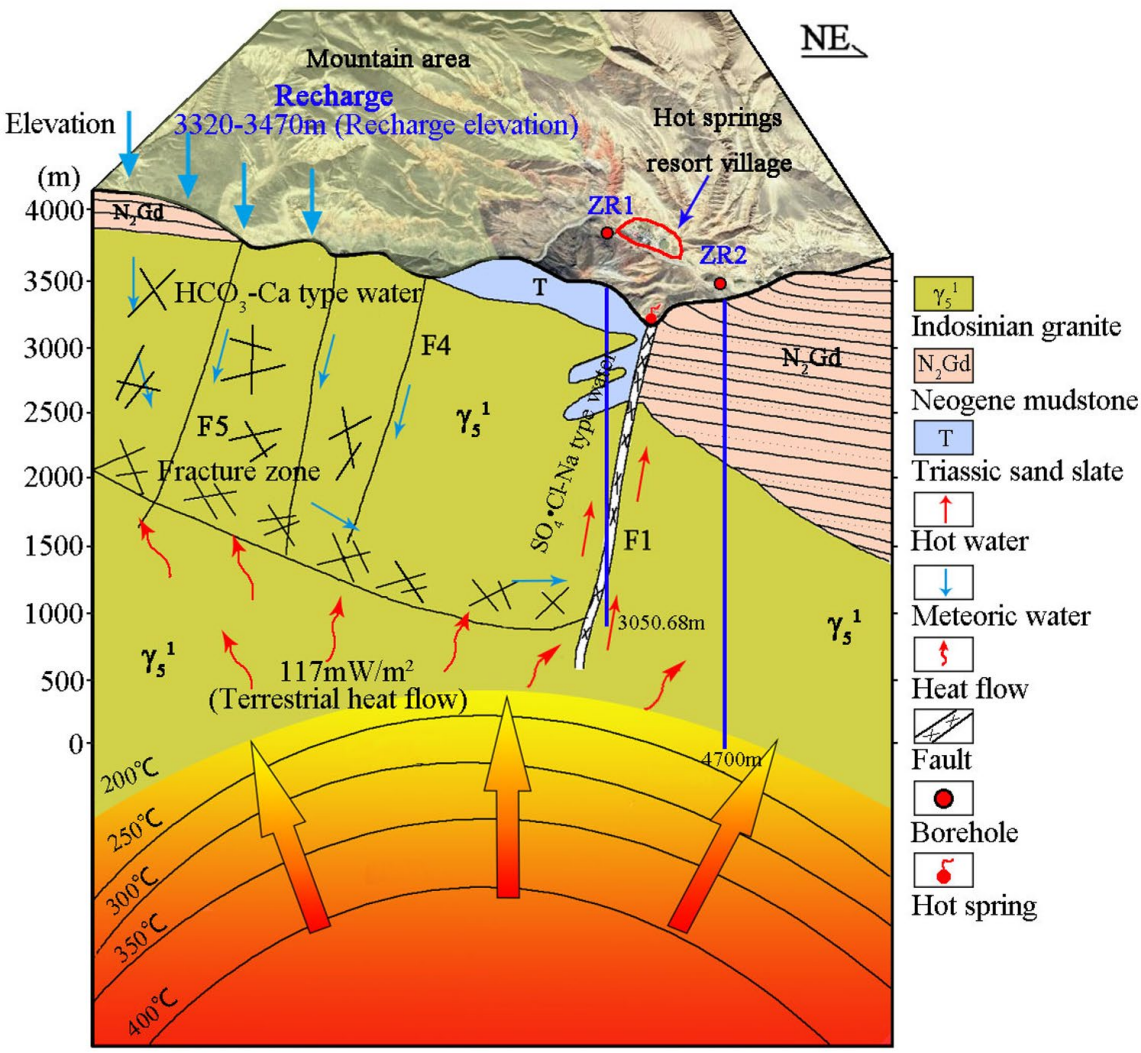
Conceptual model of the Zhacang geothermal reservoir. Figure 2 used CorelDRAW (version number: 2019, URL: https://www.coreldraw.com/cn/) to complete the geological elements. The map of Zhacang area is generated using open-access data from National Platform for Common Geospatial Information Services (Tianditu) (public domain, https://www.tianditu.gov.cn/).

The conceptual model of the Zhacang geothermal reservoir is shown in Fig. 2^34^. In general, geothermal resources have four conditions: thermal source, thermal reservoir, caprock and thermal channel. (a) Thermal source, according to the geophysical exploration and drilling data of boreholes ZR1 and ZR2, the depth and bottoming temperature are 3,050.68 m/151.34 °C and 4,721.6 m/214 °C respectively. The Zhacang geothermal field has a high geothermal heat flow value (134 *mW/m*^*2*^), the main heat source is the inner mantle heat of the earth, deep faults cut into the lithosphere connecting the deep heat source, and the asthenospheric material rises along the fault zone. (b) Thermal reservoir, the scale of the basement intrusions is large, and the lithology is mainly Indosinian granites and granodiorite, which are good high‐temperature geothermal reservoirs. (c) Caprock, the thickness of Neogene mudstone and siltstone with poor thermal conductivity and permeability is about 226.3–532 m, forming a good caprock. (d) Thermal channels, the study area contains deep and large fault zones and derived many secondary faults, which are the main passages of deep heat sources and groundwater migrating upward.

### The preliminary target reservoir

**Fig. 3:**
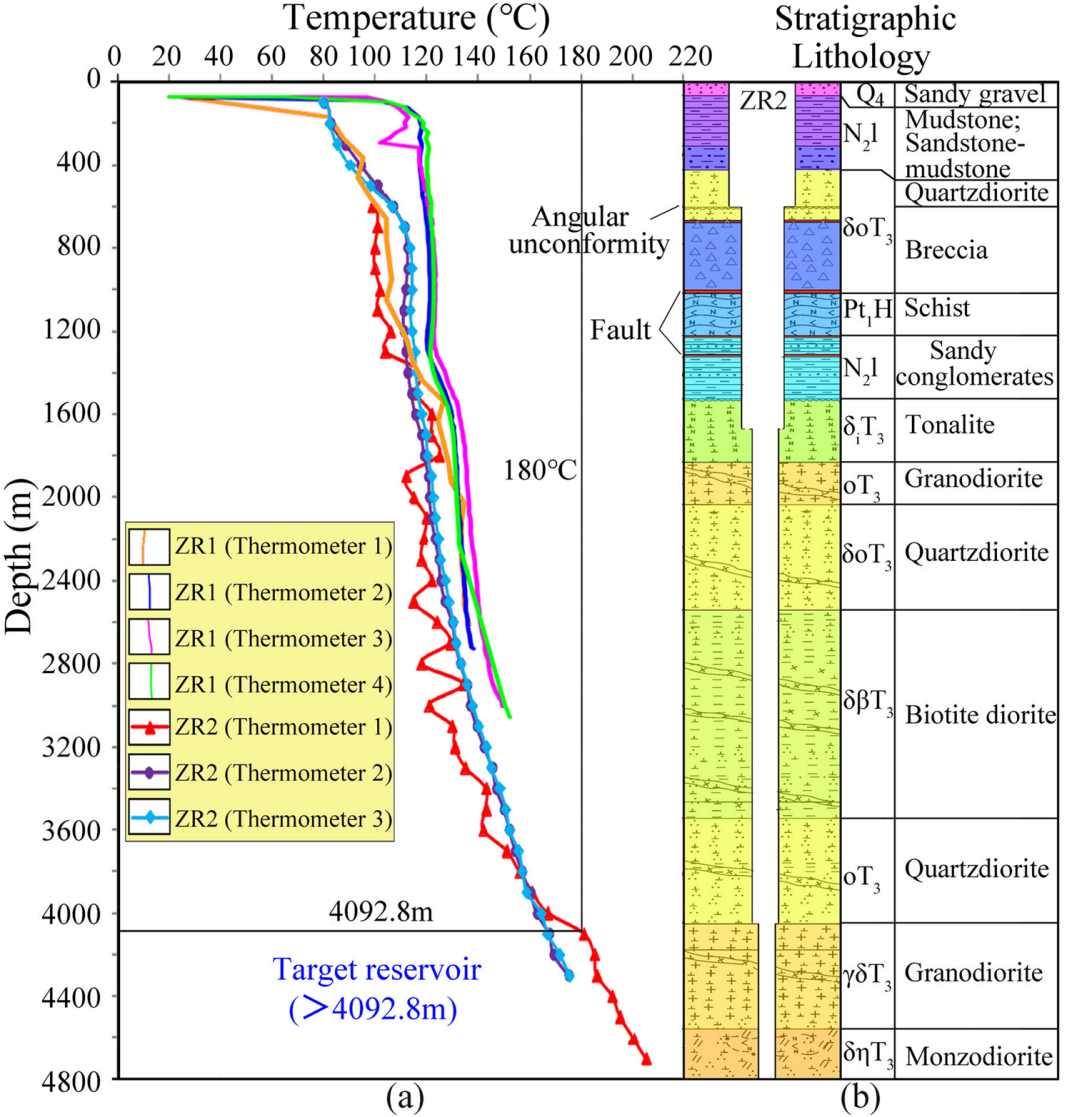
Temperature logs in thermal boreholes ZR1 and ZR2 and the lithology description in well ZR2 in the Zhacang geothermal field.

In order to explore the characteristics of high-temperature geothermal storage of Zhacang geothermal field, two geothermal boreholes ZR1 and ZR2 have been completed. Based on the temperature measurement curves obtained from these boreholes (Fig. 3), the temperature of borehole ZR1 increased from 142.1 to 151.34 ℃ within the depth range of 2700–3050 m. The temperature in borehole ZR2 is lower than that in borehole ZR1, and reached 180 °C at 4092.8 m, and the well temperature at 4602 m was 214 °C. The geothermal gradient was 4.94 ℃/100 m from 3000 to 4700 m. The borehole histogram of ZR2 is shown in Fig. 3. The sedimentary rocks with low thermal conductivity above 1500 m depth are mainly mudstone and sand-conglomerate, which have the ability of heat insulation for thermal reservoirs. The temperature of HDRs generally exceeds 180 °C, which corresponds to the stratum below the depth of 4092.8 m in this study area, and the lithology is mainly granodiorite, quartz diorite, and biotite diorite.

**Fig. 4:**
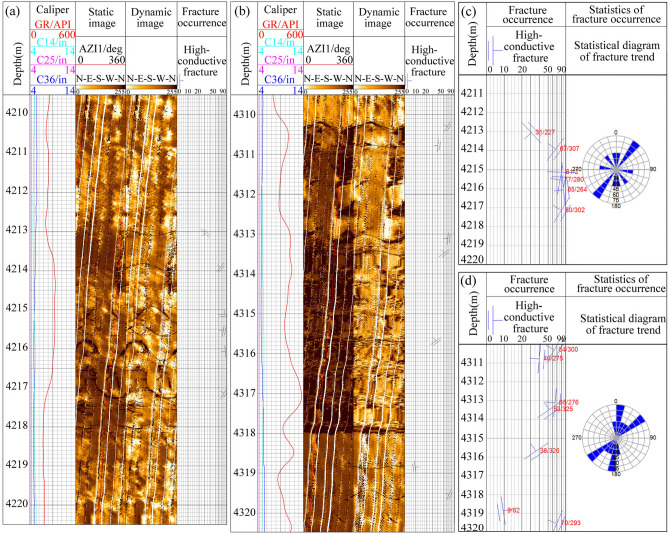
Fracture identification and occurrence statistics of electrical imaging logging in 4210-4220 m (**a**,**c**) and 4310-4320 m formations (**b**,**d**).

The Qinghai Bureau of environmental geology exploration conducted four logging operations on borehole ZR2 to investigate the characteristics of the fault crush belt and ground stress. The logging program included various measurements such as apparent resistivity, borehole diameter, acoustic wave, spontaneous potential, and ground stress. A total of 31 fractures were identified from the 3960–4518 m section of borehole ZR2 based on the image features, among which the fractures in the 4210–4220 m and 4310–4320 m sections are well developed, with 6 and 7 fractures, respectively (Fig. 4a-b). The fractures have dip angles of 30.8°-85°, inclinations of 264°-325°, widths of 0.96–2.64 mm, lengths of 1.30–4.90 m/m^2^. The fracture apertures are large and there are no mud filling, which belong to high conductivity fractures. The occurrence statistics diagrams of two fracture-developed zones are shown in Fig. 4c-d, which exhibit a similar strike direction of NNE and NE^40^. The occurrence of fractures is closely related to the in-situ stress direction. The direction of the maximum principal stress aligns with the fracture direction, which is NE-SW, consistent with the analysis results obtained from the borehole wall caving method and the three-well diameter method^41^. The high temperature of the formation indicates high thermal storage capacity, and the well-developed fractures in the formation can facilitate reservoir modification to enhance thermal conversion efficiency^29^. In the study area, the total fracture development section is 20 m, and the other sections are dense granodiorite, so it is necessary to modify the reservoir to improve the porosity and permeability to increase the production capacity. Consequently, the formation within the 4200–4400 m depth range was selected as the target reservoir to explore the optimal strategy for enhancing the power generation performance of EGS.

### Basic properties of the reservoir

**Fig. 5:**
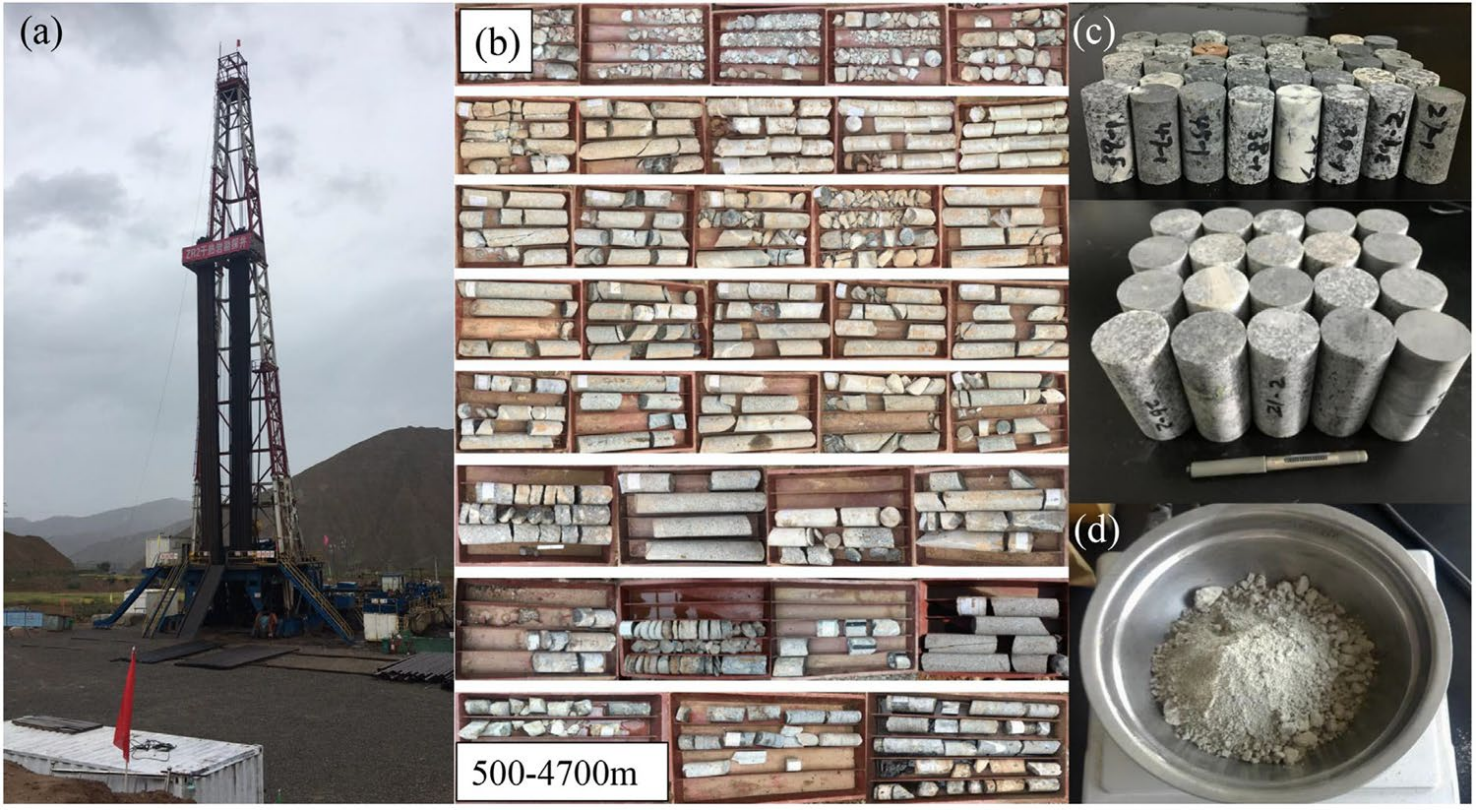
Schematic of (**a**) photo of field drilling of the ZR2 well, (**b**) the obtained drilling cores from ZR2 well, (**c**) the processed cylindrical rock specimens and (**d**) the processed powdered rock specimens.

The thermal conductivity plays a crucial role in characterizing the ability of rocks to conduct heat and is primarily used in calculating heat transfer within rocks. The specific heat capacity is an important parameter that relates to the heat storage capacity of reservoirs. Porosity refers to the extent of pore and crack development in rocks, which significantly influences their mechanical strength, deformation characteristics, and permeability. The permeability affects the distribution of fluid flow within rocks. Therefore, it is important to obtain these parameters of the reservoir for geothermal reservoir assessment, selection of fracturing methods and design of system heat generation schemes. Nearly 50 rock samples were collected from the 500–4700 m cores of borehole ZR2 in Zhacang geothermal field to carry out laboratory tests (Fig. 5). The permeability and porosity of these samples were measured using a KS-1 gas permeation tester with nitrogen based on the gas method. The Thermal Conductivity Scanner (TCS) developed by Popov was used to measure the thermal conductivity^42^, and the Bending Beam Rheometer (BBR) was used to measure specific heat capacity.

**Fig. 6:**
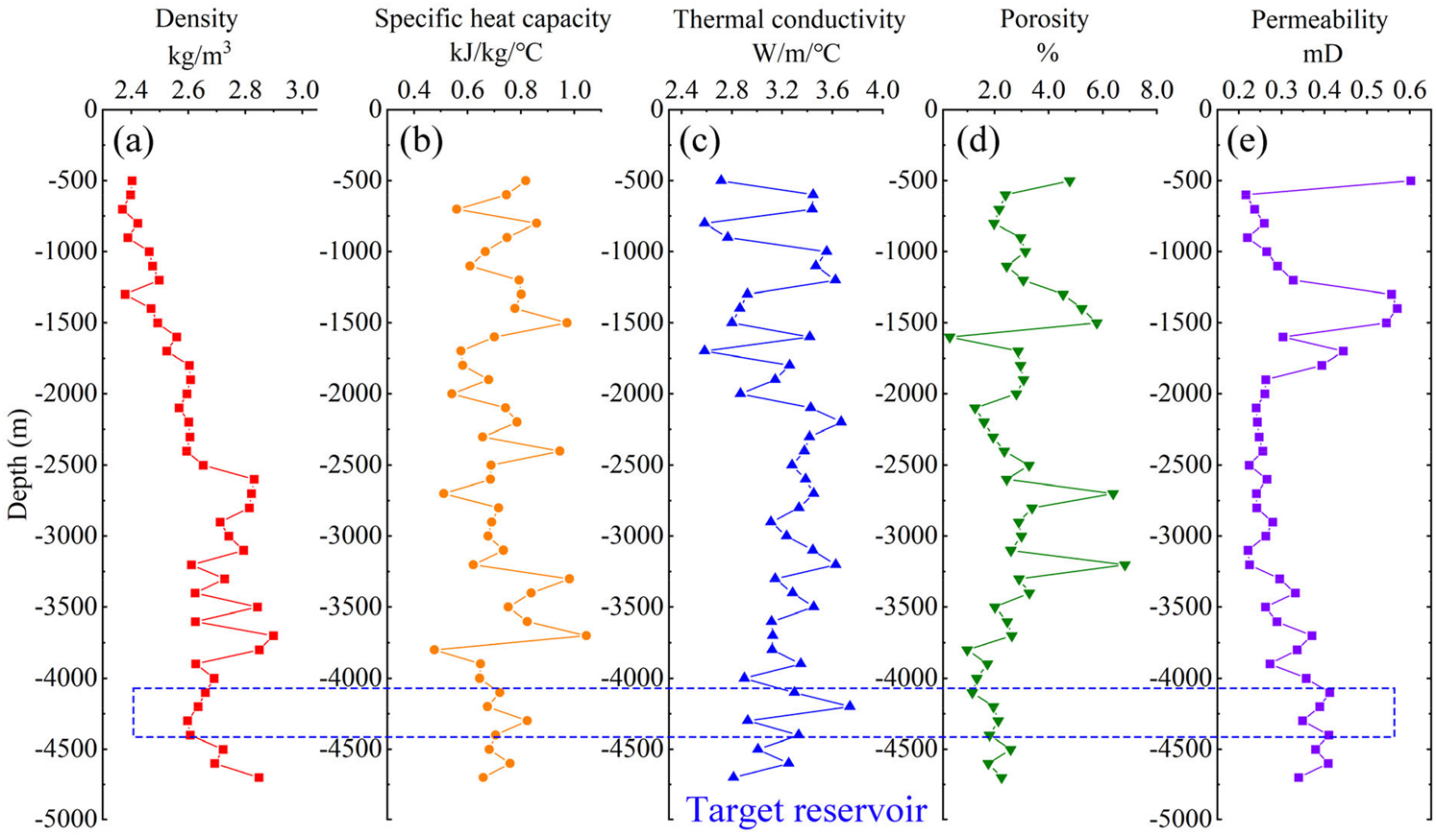
The changing curves of the thermophysical parameters of well ZR2 with depth. (**a**) density, (**b**) specific heat capacity, (**c**) thermal conductivity, (**d**) porosity and (**e**) permeability.

The laboratory test results are shown in Fig. 6. The density of core samples from the borehole ZR2 ranges from 2369 kg/m^3^ to 2899 kg/m^3^, with an average value of 2619 kg/m^3^ (Fig. 6a). The values of specific heat capacity ranged from 0.476–1.045 kJ/(kg·K), with most of the core samples having a specific heat capacity distribution between 0.6 kJ/(kg·K) and 0.8 kJ/(kg·K) (Fig. 6b). The thermal conductivity is distributed between 2.583–3.738 W/(m·K), with granitoids displaying higher thermal conductivity compared to sedimentary rocks (Fig. 6c). The porosity ranges from 0.34% to 6.82% and exhibits a decreasing trend with depth, primarily due to the rock becoming denser at greater depths (Fig. 6d). The permeability ranges from 0.216 to 0.602 mD (Fig. 6e). For the granodiorite thermal reservoir, its density is 2.613 g/cm^3^, thermal conductivity is 3.33 W/(m·K), specific heat capacity is 0.73 kJ/(kg·K), porosity is 2.0%, and permeability is 0.38 mD. In the Soultz EGS project in France, the reservoir permeability after hydraulic fracturing can reach 50 mD, so it is impossible to rely solely on natural fractures and the permeability of the reservoir for geothermal extraction in Zhacang geothermal field, and artificial fracturing is required to modify the reservoir.

## Geothermal extraction strategies

The power generation performance of an EGS is influenced by various parameters, including reservoir natural properties, completion parameters, and operational parameters. Natural properties are an inherent attribute of geothermal reservoir, the Zhacang geothermal field reservoir with a temperature greater than 190 ℃, and lithology is granodiorite with high thermal conductivity, which is a good thermal reservoir. The completion parameters can determine the type of EGS to be established, including the number and type of wells, well spacing, and the number and spacing of hydraulic fractures. Nowadays, the vertical wells and horizontal wells are common types of wells used. With the maturity of horizontal well technology in the oil and gas industry, researchers have started exploring the combination of traditional hydraulic fracturing technology and horizontal well technology. By utilizing multi-stage fracturing technology in horizontal wells, multiple fractures can be created, increasing heat production and reducing flow impedance^43^. Thus, in this study, a three-horizontal-well system is employed to evaluate the power generation potential of thermal reservoirs.

### Enhanced geothermal system design

Artificial fractures are typically created through hydraulic fracturing and chemical stimulation. Chen et al^44^. showed that the fracture permeability produced after artificial fracturing was about 1000 mD (about 0.987 × 10^–12^ m^2^) when the confining pressure is greater than 40 MPa. Many scholars have also studied hydraulic fracturing of geothermal fields in the Gonghe Basin. Lei et al^3^. simulated hydraulic fracturing in a dense granite reservoir at a depth of 3500–3705 m in the Qiabuqia geothermal field, and the fracturing process with gel proppant could produce hydraulic fractures with a half-length of 300–600 m, a fracture height of about 85 m, and an average permeability of 6.0 × 10^–11^ m^2^. Zhang et al^29^. designed a two-horizontal well fracturing scheme for granodiorite in the Zhacang geothermal field, achieving a modified fracture permeability of 114.9 D (about 1.13 × 10^–10^ m^2^) and a fracture width of 8 mm. The operating parameters include the type of working fluid, the rate and temperature of fluid injection, which determine the lifespan and heat production of EGS. Based on global EGS field experiences, the injection rate ranges from 10 to 75 kg/s, and the injection temperature ranges from 20 to 80 ℃^5,45^. Generally, higher injection rate and lower injection temperature will result in higher heat recovery rate, but it will also rapidly deplete the reservoir.

For the target reservoir in Zhacang geothermal field, the natural properties are fixed. It is important to explore the effects of completion parameters and operational parameters on each performance index to rationally assess the power generation potential of three-horizontal-wells EGS in Zhacang geothermal field. After comprehensive consideration, we selected five factors that exhibit a significant relationship with each index. These factors include well spacing, injection rate, injection temperature, fracture permeability, and fracture spacing^5,45^. However, the relationship between these factors and the power generation performance of EGS is not a simple linear relationship. To determine the optimal exploitation strategy suitable for thermal reservoirs, it is necessary to evaluate as many combinations of influencing factors as possible, which requires the establishment of millions of hydrothermal coupling numerical models. In order to simplify the workload and save the calculation time, BPNN combined with numerical simulation can be introduced to solve this problem.

### Hydrothermal simulator

In this study, the data samples used to create the BPNN model were collected from the numerical model, which was established by the EOS1 module in the TOUGH2 groundwater simulation program. TOUGH2 is a hydrothermal simulator developed at Lawrence Berkeley National Laboratory in the United States^46^. It simulates non-isothermal multicomponent flow in porous and fractured media using the integral finite difference approach. A fractured porous medium resembling a low-permeability rock matrix block embedded in a high-permeability fracture network will be created after hydraulically fractured hot dry rock reservoirs. The rock matrix and fractures exchange fluid and heat through “interporosity flow”. To describe the fluid and heat exchange in fractured reservoirs, it is important to resolve for the driving pressure, temperature, and mass fraction gradients at the matrix/fracture interface. The Multiple INteracting Continua (MINC) module in TOUGH2 can discretize porous media into subgrids to form a multi-continuum media model, which can more accurately describe the pressure changes and heat transfer between the rock matrix and fractures. The simulator is widely used in numerical studies of geothermal energy development and many studies have evaluated the accuracy of the code.

### Modeling and model conditions

**Fig. 7:**
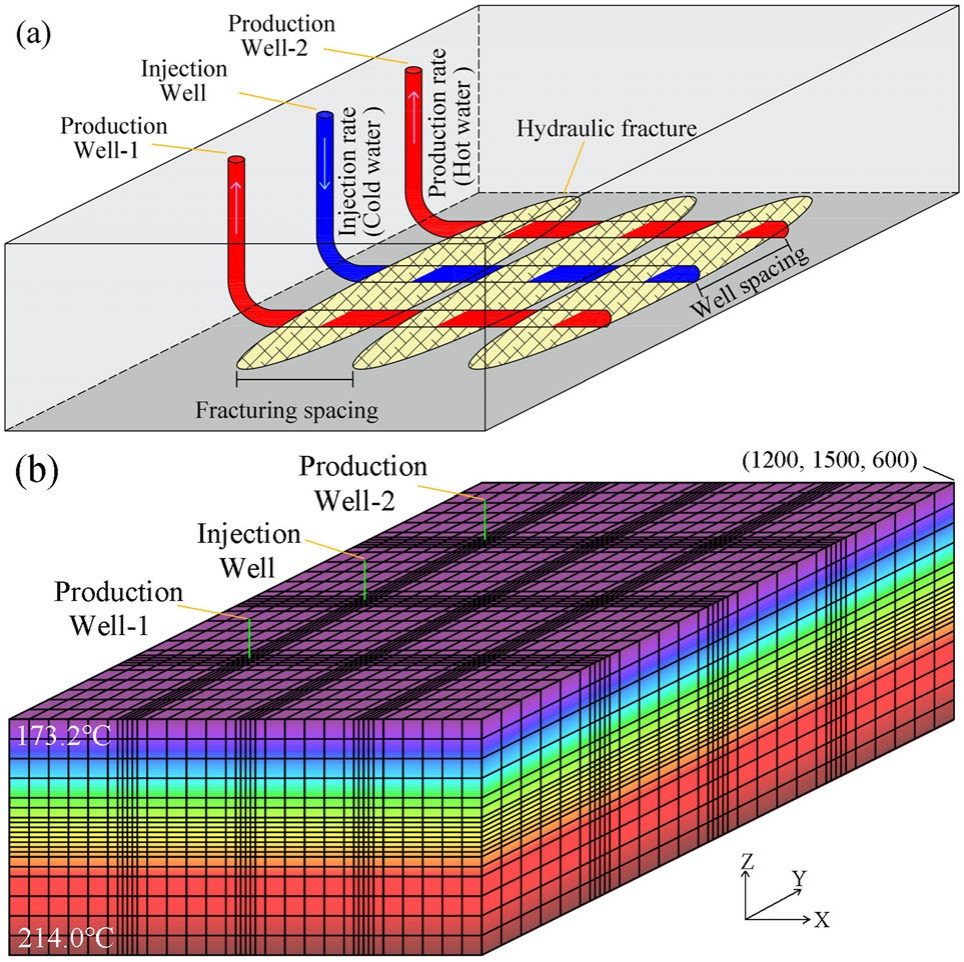
(**a**) Schematic diagram of the three horizontal wells extraction system, (**b**) 3-D view of an example reservoir model with the initial temperature distribution.

In this study, the power generation potential of the thermal reservoir is evaluated using a three horizontal wells extraction model, which is an EGS system consisting of one injection well and two production wells, with injection in the center and pumping on both sides, and a horizontal well length of 900 m, located at 4300 m below ground. The trajectory of the horizontal wells should be approximately perpendicular to the maximum horizontal stress (NE-SW) to ensure that production and injection wells can be connected through artificial fractures, which can be engineered to enhance the heat production of the thermal reservoir by using multistage hydraulic fracturing to form multiple fractures parallel to each other along the direction of the horizontal wells, as shown in the schematic diagram in Fig. 7a.

In this study, a 3D hydrothermal coupling base model of three horizontal wells is developed using TOUGH2-EOS1. The model consists of an injection well and two production wells, with a horizontal section length of 900 m and a well spacing of 500 m in the enhanced reservoir. The model with a size of 1200 × 1500 × 600 m was created at depths between 4000 and 4600 m. Based on logging data, it is observed that cracks are highly developed in the formation from 3960 to 4518 m, particularly at 4200–4400 m, making it suitable for hydraulic fracturing. Previous research on hydraulic fracturing in thermal reservoirs has shown that hydraulic fracture heights range from 100 to 600 m, fracture widths range from 2 to 8 mm, and permeabilities range from 1 × 10^–12^ m^2^ to 1 × 10^–10^ m^2^^29,40^. For this study, it is assumed that the enhanced thermal reservoir (4200–4400 m) has a fracture height of 200 m, a width of 2 mm, a fracture spacing of 200 m, and a fracture permeability of 1 × 10^–11^ m^2^. The enhanced reservoir domains are defined as 150 < x < 1050 m, 150 < y < 1350 m and 200 < z < 400 m. The model, depicted in Fig. 7b, is discretized into a total of 23,562 cells. To increase the accuracy of the simulation results, the grid is refined close to the injection wells, production wells, and reservoirs. The initial reservoir temperature distribution of the model is based on the temperature logging curve of the ZR2 well, and the initial reservoir pressure is calculated according to P = 4.6 × 10^7^- 10000z(Pa). To ensure stable heat production, the 50 °C water is injected cyclically at 20 kg/s into the enhanced reservoir. The model boundary is set as a closed boundary without mass and heat transfer, considering that although the dense surrounding rock with low permeability outside the enhanced reservoir can recharge heat to the thermal reservoir by heat transfer, the rate is very slow and the exchange is very small. The detailed parameters of this geothermal mining base simulation are listed in Table 1, where the reservoir rock thermal properties linked to heat exchange were determined from laboratory testing. The simulation is conducted for a period of 50 years. Based on the base model, a total of 3^2^ × 4^3^ = 576 simulated working conditions were set up by applying different parameter conditions according to Table 2.

**Table 1:** Reservoir properties of the base model.

Parameters	Value
Rock density	2613 kg/m^3^
Rock porosity	2.0%
Rock permeability(kx = ky = kz)	3.80 × 10^–16^ m^2^
Rock thermal conductivity	3.33 W/(m⋅K)
Rock specifific heat capacity	730 J/(kg⋅K)
Fracture porosity	50%
Fracture permeability, (kx = ky = kz)	1.0 × 10^–11^ m^2^
Initial reservoir temperature	T = 214.0 − 0.068z (℃)
Initial reservoir pressure	P = 4.6 × 10^7−^10000z (Pa)
Productivity index	5.0 × 10^–12^ m^3^
Bottom-hole production pressure	42 MPa
Operation time	50 years

**Table 2:** The values of the five key factors.

	Value			
Well spacing, (m)	400	500	600	
Water injection tate, (kg/s)	10	20	25	30
Injection temperature, (℃)	20	50	65	80
Fracture permeability, (m^2^)	1 × 10^–12^	1 × 10^–11^	1 × 10^–10^	
Fracturing spacing, (m)	100	150	200	300

### Performance criteria

The general requirements for geothermal extraction are as follows: having a high production temperature, high production, long mining time and high degree of extraction. By summarizing the previous literature, the following were selected as geothermal evaluation indicators to comprehensively evaluate power generation performance, the 50th year production temperature ($T_{50}$), heat extraction efficiency ($R$), flow impedance ($I_{R}$), electric power ($W_{e}$) and thermal efficiency($\eta_{th}$).

(1) $T_{50}$ is the production temperature of the system up to the 50th year of operation, which reflects the thermal performance of the geothermal system, ℃. $T_{pro}$ is the production temperature, ℃. In order to ensure stable power generation of the system, the production temperature drop within the designed operating life should not exceed 10%^47^.

(2) The heat extraction efficiency ($R$) can directly reflect the heat recovery capacity of the system over a period of time^37^. The heat extraction rate is defined as the ratio of the extracted thermal energy to the total thermal energy in the thermal storage within 50 years of system operation.

1 $$R = \frac{{\int_{0}^{{\Gamma_{50} }} {W_{h} \left( t \right){\text{d}}t} }}{{V\left( {T_{r} - T_{inj} } \right) \cdot \left[ {\left( {1 - n} \right)\rho_{r} c_{r} + n\rho_{w} c_{w} } \right]}} = \frac{{\int_{0}^{{\Gamma_{50} }} {2q\left[ {\left( {h_{pro} \left( t \right) - h_{inj} } \right)} \right]{\text{d}}t} }}{{V\left( {T_{r} - T_{inj} } \right) \cdot \left[ {\left( {1 - n} \right)\rho_{r} c_{r} + n\rho_{w} c_{w} } \right]}}$$

where $W_{h} (t)$ is the heat extraction efficiency of the system at time t^48^, kW; $\Gamma_{50}$ is the total operating time, 50 years; $V$ is the volume of reservoir, m^3^; $T_{r}$ is the temperature of the rock reservoir, ℃; $T_{inj}$ is the injection temperature, ℃; n is the rock porosity; $\rho_{r}$ and $\rho_{w}$ are reservoir rock density and fluid density, kg/m^3^; $c_{r}$ and $c_{w}$ are the specific heat capacity of the rock reservoir and the specific heat capacity of the fluid, kJ/(kg·℃); $q$ is the output flow rate of an individual production well, kg/s; $h_{pro}$ is the specific enthalpy of effluent, $h_{pro} = h(T_{pro} ,P_{pro} )$, kJ/kg; $h_{inj}$ is the specific enthalpy of water injection, kJ/kg.

(3) The flow impedance ($I_{R}$) is the amount of energy consumed to drive a unit mass of fluid through a thermal reservoir, reflecting the ease with which the fluid can flow through the thermal reservoir. $I_{R}$ is the average flow impedance of EGS^49^.

2 $$I_{R} = \frac{{\int_{0}^{{\Gamma_{50} }} {I_{R} \left( t \right){\text{d}}t} }}{{\Gamma_{50} }} = \frac{{\int_{0}^{{\Gamma_{50} }} {{{P_{inj} \left( t \right) - P_{pro} \left( t \right)} \mathord{\left/ {\vphantom {{P_{inj} \left( t \right) - P_{pro} \left( t \right)} q}} \right. \kern-0pt} q}{\text{d}}t} }}{{\Gamma_{50} }}$$

where $P_{inj}$ is the injection pressure, MPa. $P_{pro}$ is the bottom-hole pressure of the production well, MPa.

(4) $W_{e}$ is the average electrical power of EGS. According to the laws of thermodynamics, the proportion of total heat production that can be converted to maximum mechanical work is $f$, and the utilization efficiency of maximum mechanical work that can be converted to electricity is 0.45^43^.

3 $$W_{e} = \frac{{\int_{0}^{{\Gamma_{50} }} {W_{e} \left( t \right){\text{d}}t} }}{{\Gamma_{50} }} = \frac{{\int_{0}^{{\Gamma_{50} }} {0.45fW_{h} \left( t \right){\text{d}}t} }}{{\Gamma_{50} }}$$

4 $$f = 1 - T_{rej} /Tpro$$

where $T_{rej}$ is the heat rejection temperature, with an annual average temperature of 7.2 ℃ and an exhaust temperature of 280.35 K in the Guide Basin.

(5) The calculation formula for the average EGS thermal efficiency ($\eta_{th}$) within 50 years is as follows^50^:

5 $$\eta_{th} = \frac{{W_{e} - W_{p} }}{{W_{h} }}$$

6 $$W_{p} = \frac{{\int_{0}^{{\Gamma_{50} }} {W_{p} \left( t \right){\text{d}}t} }}{{\Gamma_{50} }} = \frac{2q}{{\rho \eta_{p} \Gamma_{50} }}\int_{0}^{{\Gamma_{50} }} {\left[ {P_{inj} \left( t \right) - P_{pro} \left( t \right) + \rho gh_{2} - \rho gh_{1} } \right]} {\text{d}}t$$

where $W_{p}$ is the average internal energy consumption of the EGS, which is the sum of the energy consumption of the injection and production pumps, kW; $W_{h}$ is the average heat recovery rate of EGS, kW; $\rho$ is the water density, kg/m^3^; $\eta_{p}$ is the efficiency of the pump, in this study, $\eta_{p} = 80\%$^51^; $g$ is the acceleration of gravity, m/s^2^; $h_{1}$ is the depth of the injection well, and $h_{2}$ is the depth of the production well, $h_{1} = h_{2} = 4250m$.

In conclusion, a total of 576 datasets of working conditions were established in this study, which consisted of five key influenced parameters and the corresponding five performance indicators. The former is used as the input set of the BPNN model and the latter is the output set of the BPNN model.

## PSO-GA-BPNN model of EGS

BPNN is a well-known neural network model that consists of an input layer, hidden layer(s), and an output layer. However, BPNN training and learning processes can encounter challenges such as local minima and slow convergence. Particle Swarm Optimization (PSO) algorithm is a population intelligence algorithm designed by simulating the predation behavior of birds^52^. It utilizes the interaction of information between individuals to continuously optimize the search process, offering advantages such as high fitting accuracy and shorter identification time. However, it has some limitations, including premature convergence and a tendency to fall into local extreme values, which is mainly due to the loss of population diversity in the search space. The cross-mutation operation of Genetic Algorithm (GA) can better ensure population diversity^53^. GA is an adaptive global optimization algorithm that simulates the genetic and evolutionary processes of organisms in a natural environment. It excels in solving nonlinear and multi-objective function optimization problems and demonstrates clear advantages in solving complex combinatorial optimization problems. GA algorithm has stronger global search ability but relatively complex process, while PSO algorithm has relatively simple operation process and faster convergence speed. In view of their complementary advantages, the two algorithms are combined to form PSO-GA-BPNN model^54^. In this paper, the PSO-GA-BPNN model is applied to predict the temperature, enthalpy difference and pressure difference in EGS for efficient and accurate estimation of EGS power generation capacity in three horizontal wells.

### The steps of PSO-GA-BPNN

**Fig. 8:**
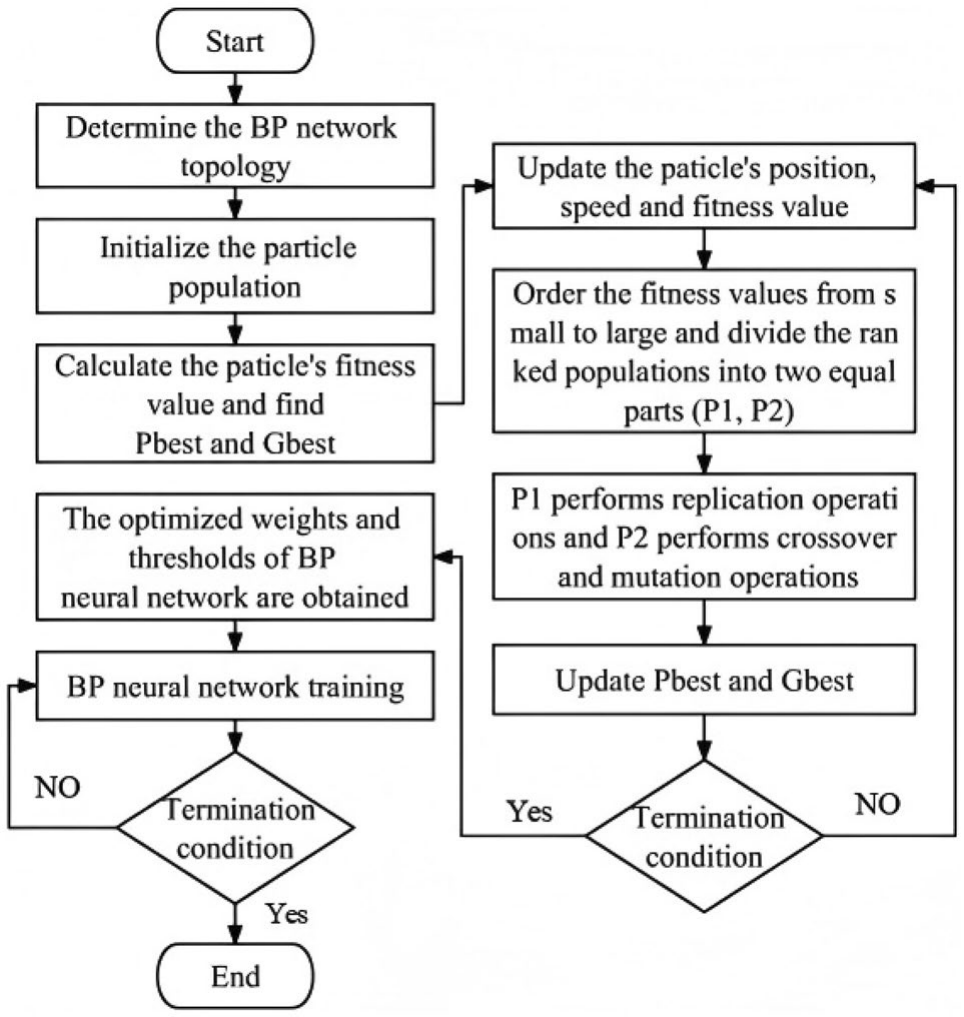
The implementation steps of PSO-GA-BPNN.

PSO-GA-BPNN model aims to optimize the weight and threshold of BPNN through PSO and GA. Combining the advantages of the two algorithms, the fusion algorithm has faster convergence speed, better global convergence performance, less computation and high robustness. The implementation steps of PSO-GA-BPNN are shown in Fig. 8.

(1) Initialize the parameters required by particle population, particle swarm algorithm and genetic algorithm.

(2) Calculate the fitness value of the particle, and compare the optimal fitness values to find out the best position of the individual $P_{best}$ and the best global position $g_{best}$ of the particle swarm.

(3) Use the PSO algorithm to constantly update the position and speed of individuals in the population, as shown in Eq. 7 and Eq. 8^55^:

7 $$v_{ij} \left( {k + 1} \right) = \omega v_{ij} \left( k \right) + c_{1} r_{1} \left( {P_{ij} \left( k \right) - x_{ij} \left( k \right)} \right) + c_{2} r_{2} \left( {P_{gj} \left( k \right) - x_{ij} \left( k \right)} \right)$$

8 $$x_{il} \left( {k + 1} \right) = x_{il} \left( k \right) + v_{il} \left( {k + 1} \right)$$

where $v$ is the particle velocity; $x$ is the particle position; $i$ is the i-th particle, $j$ is the dimension of the particle; $k$ is the current number of iterations; $P_{i}$ and $P_{g}$ are the individual optimal position and the group optimal solution of particle $i$ respectively; $\omega$ is the inertia weight; $c_{1}$ and $c_{2}$ are learning factors; $r_{1}$ and $r_{2}$ are random values. The populations whose fitness values are arranged from small to large are divided into $P_{1}$ and $P_{2}$, with $P_{1}$ having good fitness and $P_{2}$ having poor fitness.

(4) Genetic algorithm was introduced to directly copy subpopulation $P_{1}$ with good fitness to the next generation, and random crossover and mutation operations were carried out on subpopulation $P_{2}$ with poor fitness.

(5) Compare the calculated new fitness value, the fitness value $P_{best}$ of the individual extreme value and the fitness value $g_{best}$ of the population extreme value, and update the individual extreme value $P_{best}$ and the global extreme value $g_{best}$ of the population.

(6) Repeat steps (2) to (5). If the best fitness value reaches the convergence accuracy or the number of iterations reaches the set maximum number, the iteration terminates and the best position of the particle swarm is taken as the parameter of the BP neural network. Otherwise, repeat the iterative operation.

At the end of PSO-GA, the optimal solution is output as the weight and threshold of BP neural network, then the neural network is retrained, and then the simulation experiment is carried out to obtain the output of the prediction function of BP neural network.

### Build PSO-GA-BPNN model

In this study, BPNN model is employed to predict the production temperature of EGS, and one BPNN model can predict the production temperature of one time node. To establish these models, 28 representative time points are selected from the 50-year system operation period. The intermediate values of the nodes are then interpolated. The BPNN consists of three layers: the input layer, hidden layer, and output layer. The input layer comprises five nodes representing the five factors that influence the heat generation ability of EGS. The output layer consists of a single node that represents the production temperature obtained through numerical simulation. The number of nodes in the hidden layer is typically determined using an empirical formula. For this model, the calculated number of hidden layer nodes is 11. The PSO-GA-BPNN model was programmed on MATLAB software to implement the required functions. The required parameters were set according to the convergence of the program results as follows: the learning rate of the BP algorithm was taken as 0.01, the accuracy of the training model was 1 × 10^–6^, the maximum number of iterations was 1,000, and the training function was the *trainlm* function. In the PSO algorithm, the population size is 20, the number of iterations is 50, and the learning factor $c_{1} = c_{1} = 2$. In the GA algorithm, the population size is 20, the number of iterations is 50, the crossover probability is 0.8, and the variance probability is 0.2.

In order to ensure sufficient training samples, 3^2^ × 4^3^ = 576 sets of numerical simulation samples were designed for BPNN model training. In order to improve the robustness of the network, 403 sets of data were selected as training samples by random function, and the remaining 173 sets were used as test samples to test the accuracy of the BPNN model. In order to improve the training speed and accuracy of the BPNN model, the following formula (Eq. 9) is used to normalize the data involved in the modeling, and then the output data is de-normalized after fitting^56^. In addition, in order to obtain other performance metrics of the system, it is necessary to predict the enthalpy difference ($h_{pro} - h_{inj}$) and pressure difference ($P_{inj} - P_{pro}$) at the production and injection wells, by the neural network and calculate the heat extraction efficiency, flow impedance, electric power and thermal efficiency according to Eqs. (1), (2), (3), (4), (5), (6).

9 $$X^{*} = \frac{{X - X_{\min } }}{{X_{\max } - X_{\min } }}$$

where $X^{*}$ denotes the standardized data samples; $X$ is the data samples; $X_{\max }$ and $X_{\min }$ means the maximum and minimum values of data samples, respectively.

### Error analysis the PSO-GA-BPNN models

In order to measure the prediction effectiveness of BPNN, three indices of the Mean Absolute Error ($MAE$), the Mean Absolute Percentage Error ($MAPE$) and the Root Mean Squared Error ($RMSE$) are used to evaluate the network performance^57^, where $y$ represents the actual value and $y{\prime}$ represents the predicted value. The calculation formulas are as follows:

10 $$MAE = \frac{1}{n}\sum\limits_{i = 1}^{n} {\left| {y_{i}{\prime} - y_{i} } \right|}$$

11 $$MAPE = \frac{100\% }{n}\sum\limits_{i = 1}^{n} {\left| {\frac{{y_{i}{\prime} - y_{i} }}{{y_{i} }}} \right|}$$

12 $$RMSE = \sqrt {\frac{1}{n}\sum\limits_{i = 1}^{n} {\left( {y_{i}{\prime} - y_{i} } \right)^{2} } }$$

**Fig. 9:**
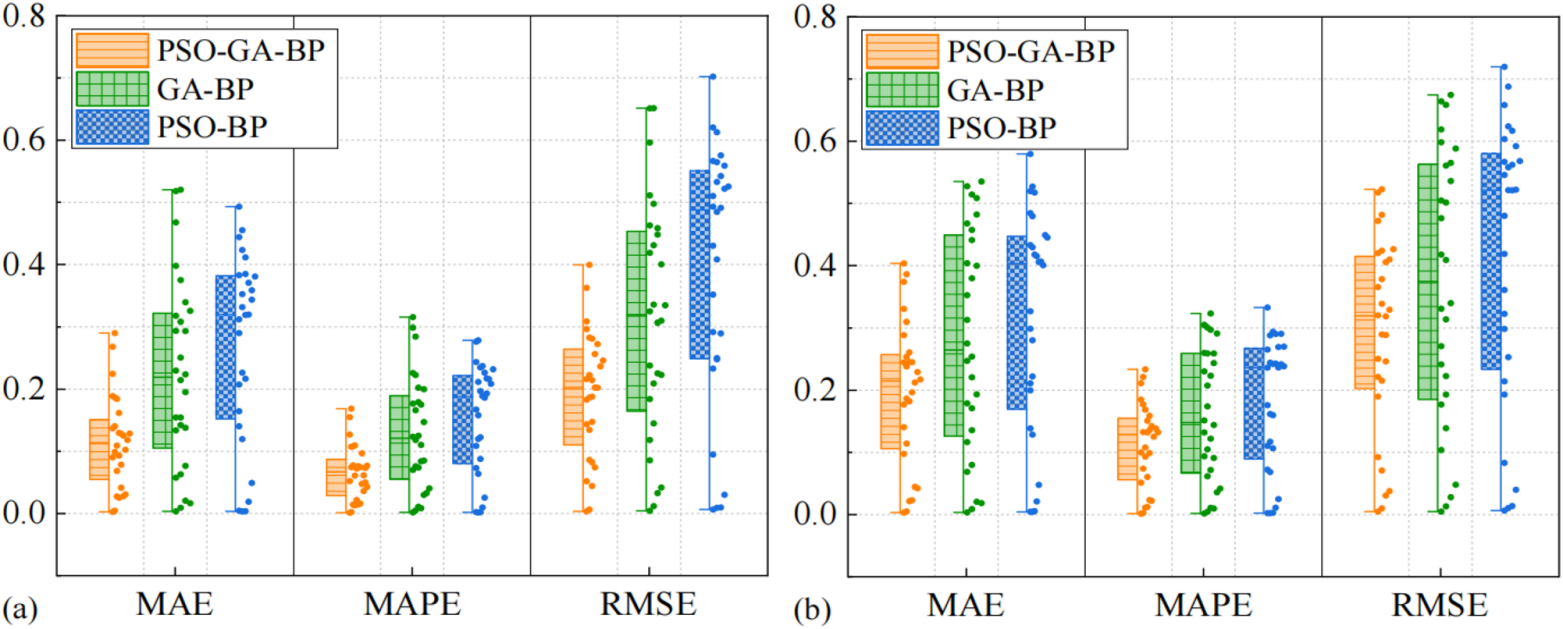
Prediction performance of different models (**a**) training data set, (**b**) the test set.

To confirm the effectiveness of the optimization of the prediction model, the PSO-GA-BPNN model, GA-BP model, and PSO-BP model were used to predict the production temperature. The comparison of the prediction results is shown in Fig. 9. The results clearly indicate that the PSO-GA-BPNN model outperforms the other models in terms of accuracy. The average MAE of the training data and the test data set in the PSO-GA-BPNN models are 0.115 and 0.196, respectively. These values are 0.103 and 0.147 smaller than those of the GA-BP models and the PSO-BP models. Similarly, the MAPE for the training data set and the test data set in the PSO-GA-BPNN models are 0.065 and 0.111, respectively. These values are 0.060 and 0.084 smaller than those of the GA-BP model and the PSO-BP model. Additionally, the average RMSE for the training data set and the test data set in the PSO-GA-BPNN models are 0.191 and 0.289, respectively. These values are 0.110 and 0.186 smaller than those of the GA-BP model and the PSO-BP model. These results show that the PSO-GA-BPNN model has better predictive ability for production temperature prediction.

**Fig. 10:**
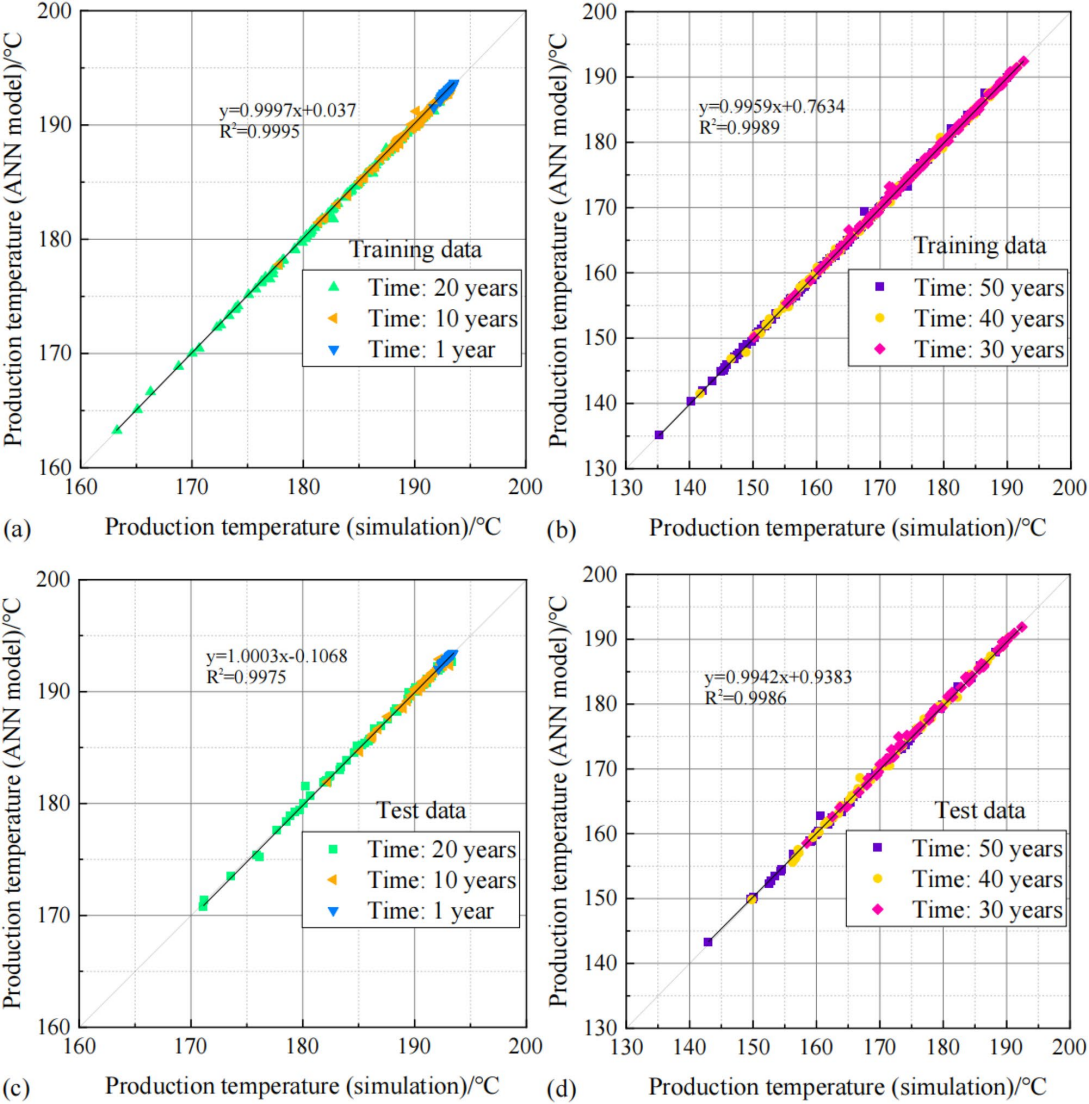
Comparison of training data with that obtained from the PSO-GA-BPNN model (**a**,**b**) training data set, (**c**,**d**) the test data set.

The fitted plot of predicted and expected values after training of PSO-GA-BPNN models are shown in Fig. 10. The solid lines are the regression lines of all datasets comprising three time nodes. The solid and 45-degree line largely overlap, with most of the data points concentrated around the solid line, indicating a good linear fit between the simulated and predicted values. The regression line slopes of 1 year, 10 years, 20-years and 30 years, 40 years, 50 years training data were 0.9997 and 0.9959, respectively, and the determination coefficients (R^2^) were 0.9995 and 0.9989, respectively. The regression line slopes of the corresponding time of the test data are 1.0003 and 0.9942, respectively, and the determination coefficients are 0.9975 and 0.9986, respectively.

This result indicates that the training quality of the model is satisfactory, and no serious inaccuracy or wrong samples are observed. Table 3 shows that the PSO-GA-BPNN model predicts the production temperature ($T_{pro}$), enthalpy difference ($h_{pro} - h_{inj}$) and pressure difference ($P_{inj} - P_{pro}$) with low values of MAE, MAPE and RMSE, indicating that it has high computational accuracy and good generalization ability. This method can reduce the calculation time of the model and increase the optimization efficiency of EGS.

**Table 3:** The predictive performance of PSO-GA-BPNN models.

	Training data set			Test data set		
	$MAE$	$MAPE$	$RMSE$	$MAE$	$MAPE$	$RMSE$
Production temperature, ($T_{pro}$)	0.115 ℃	0.065	0.191 ℃	0.1965 ℃	0.111	0.289 ℃
Enthalpy difference, ($h_{pro} - h_{inj}$)	0.487 kJ/kg	0.088	0.579 kJ/kg	0.987 kJ/kg	0.178	1.334 kJ/kg
Pressure difference, ($P_{inj} - P_{pro}$)	0.353 MPa	1.704	0.416 MPa	0.575 MPa	2.730	0.751 MPa

### Sensitivity analysis of combination factors

In this study, the previously trained PSO-GA-BPNN models are utilized to investigate the effects of pairwise parameter variations in the influencing factors on various performance indicators of the three horizontal wells EGS. The parameters being examined include well spacing (400–600 m), injection rate (10–30 kg/s), injection temperature (20–80 °C), fracture permeability (1 × 10^–12^-1 × 10^–10^ m^2^), and fracture spacing (100–300 m), as well as their interactions.

#### Production temperature

**Fig. 11:**
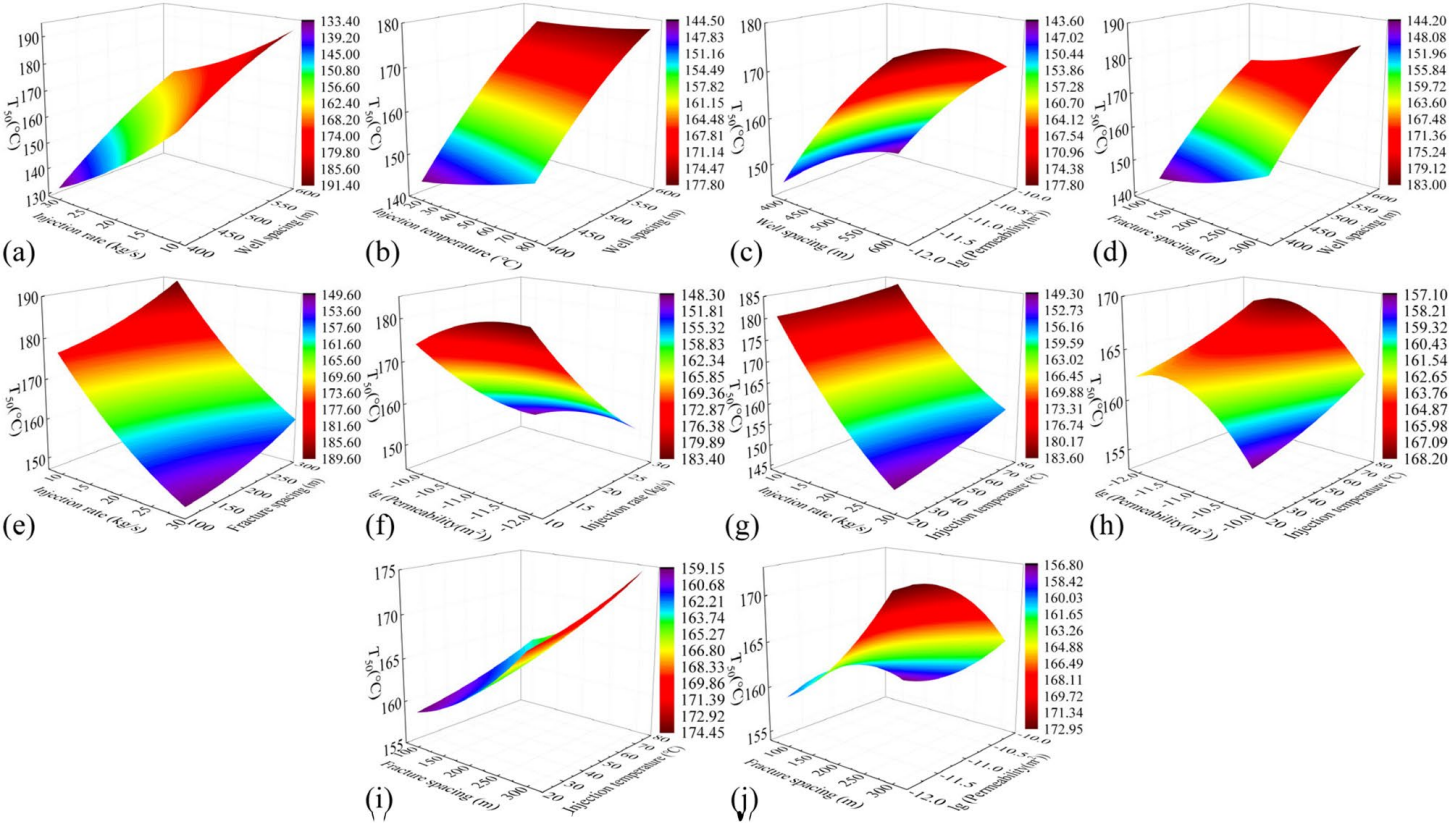
Cloud plot of the production temperature variation in the 50th year $T_{50}$ of the EGS under the influence of combined factors.

Figure 11 shows cloud plots depicting the variation of production temperature in the 50th year ($T_{50}$) under the influence of combined factors. In Fig. 11j, the steep slope and high degree of contour shape curvature indicate a significant interaction between fracture permeability and fracture spacing. Across all the plots, $T_{50}$ difference caused by different well spacing ranges from 28.8 to 33.2 ℃. The production temperature increases with increasing well spacing, as longer flow paths facilitate heat exchange between the fluid and the hot rocks in the reservoir. $T_{50}$ difference caused by injection rate and injection temperature is 31.0–34.1 °C and 5.0–7.9 °C, respectively. This is because injecting low-temperature fluid into the reservoir at a high speed accelerates temperature reduction and heat recovery in the reservoir. Both fracture spacing and fracture permeability exhibit nonlinear relationships with production temperature. $T_{50}$ difference caused by fracture spacing and fracture permeability is 10.7–13.3 °C and 5.7–8.5 °C, respectively. The production temperature decreases with increasing fracture permeability, as higher permeability leads to faster water flow in the reservoir, reducing the time available for heat exchange between the fluid and the thermal reservoir. The production temperature increases with the decrease of the fracture spacing. This increase becomes more pronounced when the fracture spacing is less than 150 m, as a smaller spacing implies more reservoir fractures and a larger heat transfer area for water flow and heat reservoir. After comparing all the factors, the main influences on production temperature were ranked as follows: injection rate, well spacing, fracture spacing, fracture permeability, and injection temperature.

#### Heat extraction efficiency

**Fig. 12:**
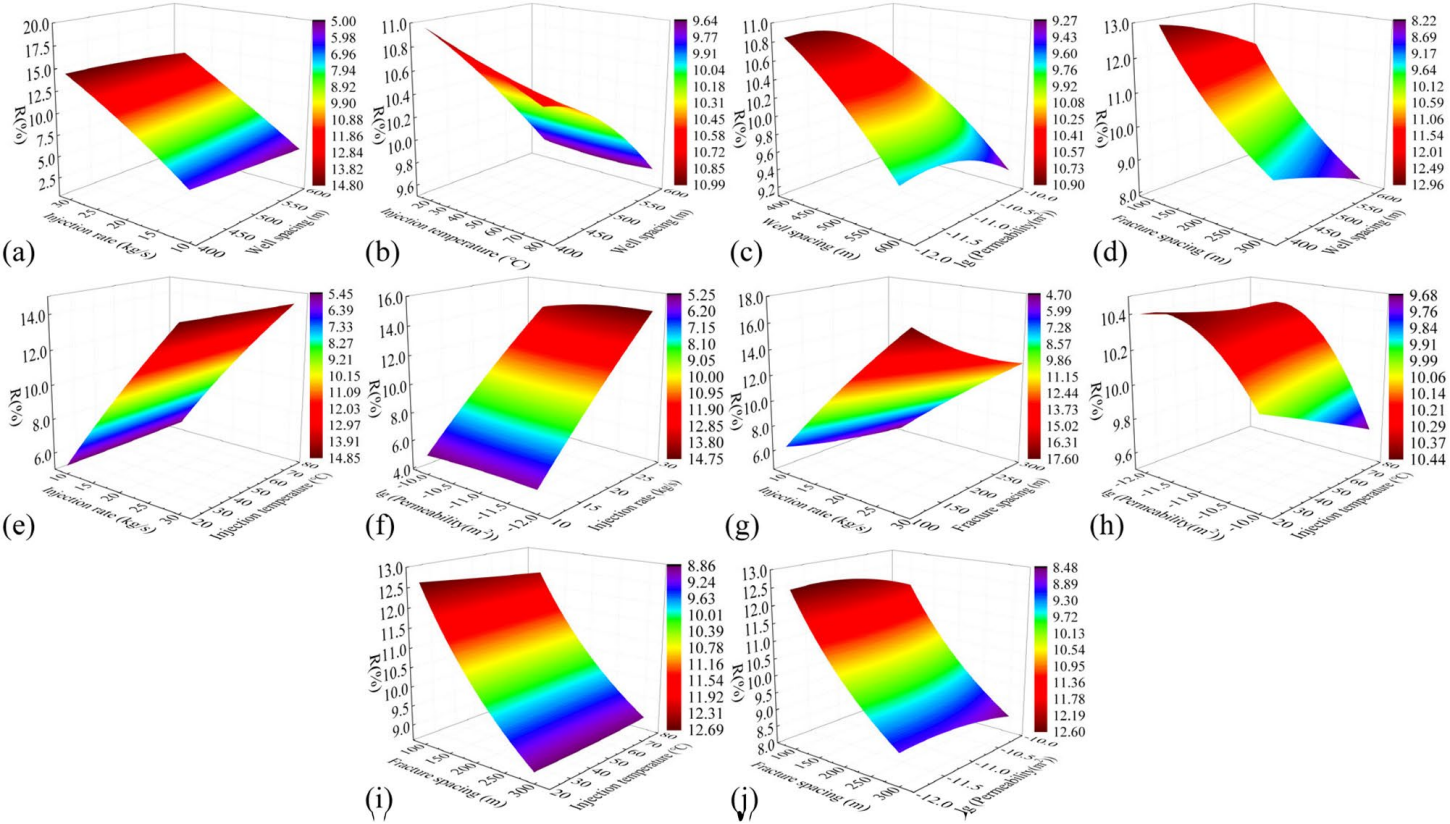
Cloud plot of the heat extraction efficiency $R$ under the influence of combined factors.

Figure 12 depicts cloud plots illustrating the variation of heat extraction efficiency ($R$) under the influence of combined factors. The surface shape of Fig. 12(c, h) is similar to the bell shape, indicating that the interaction of the two factors has a great influence on the heat extraction rate. Injection rate, fracture spacing, well spacing, and injection temperature show nearly linear relationships with $R$. For the same thermal storage volume, increasing the injection rate, decreasing the fracture spacing, and reducing the injection temperature can enhance $R$ by approximately 9.2–10.9%, 3.6–4.9%, and 0.3–0.5%, respectively. The volume of the reservoir varies with well spacing, and a smaller reservoir volume corresponds to a greater degree of geothermal resource development over the same time period. Consequently, $R$ increases with decreasing well spacing, with an approximate increase of 0.9–1.3%. $R$ also increases with decreasing fracture permeability, with an increase of about 0.7%, and the increase of heat recovery rate is not obvious when the fracture permeability is less than 1 × 10^–11^ m^2^. After comparing all the factors, the main influences on $R$ were ranked as follows: injection rate, fracture spacing, well spacing, fracture permeability, and injection temperature, in that order.

#### Flow impedance

**Fig. 13:**
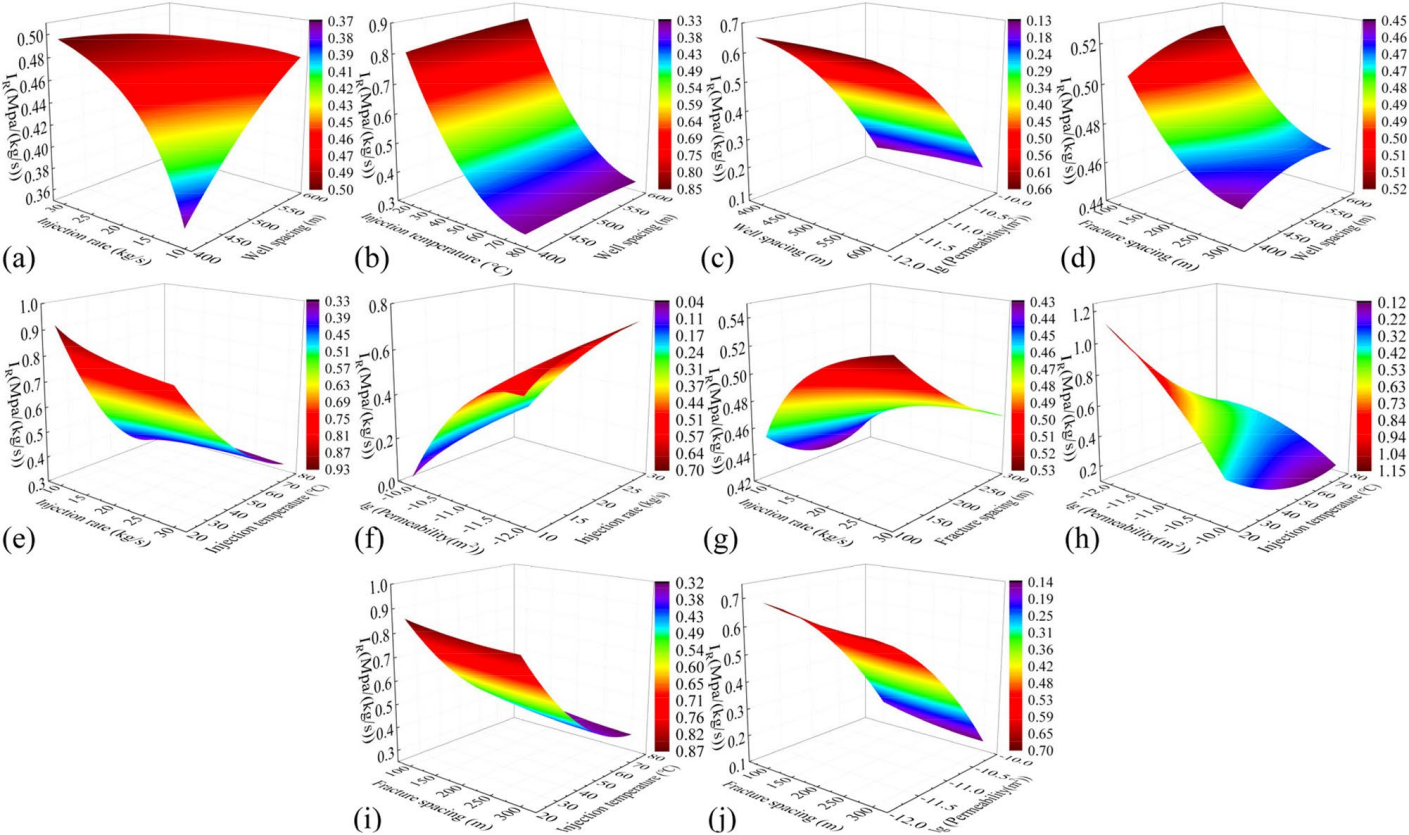
Cloud plot of the flow impedance $I_{R}$ under the influence of combined factors.

Figure 13 presents cloud plots illustrating the variation of flow impedance ($I_{R}$) under the influence of combined factors. The Fig. 13h reveals that the interaction between fracture permeability and injection temperature has a relatively significant effect on $I_{R}$. $I_{R}$ slightly increases with an increase in fracture spacing by approximately 0.06 Mpa/(kg/s). This is because a larger fracture spacing implies fewer heat exchange channels, leading to an increase in injection pressure. $I_{R}$ decreases significantly with increasing fracture permeability. The decline curve approximates a downward curved parabola, with a decrease of approximately 0.51–0.76 Mpa/(kg/s). This is because higher fracture permeability facilitates fluid flow in the reservoir, resulting in effective reduction of flow impedance. $I_{R}$ decreases by about 0.6 Mpa/(kg/s) with an increase in injection temperature. The decline curve approximates an upward curved parabola. This is because high-temperature fluids have lower viscosity, which reduces flow resistance in the reservoir. Reducing the injection rate can significantly decrease $I_{R}$ by approximately 0.08–0.17 Mpa/(kg/s). The influence of well spacing on $I_{R}$ is not significant. After comparing all the factors, the main influences on flow impedance were ranked as follows: fracture permeability, injection temperature, injection rate, fracture spacing, and well spacing.

#### Electrical power

**Fig. 14:**
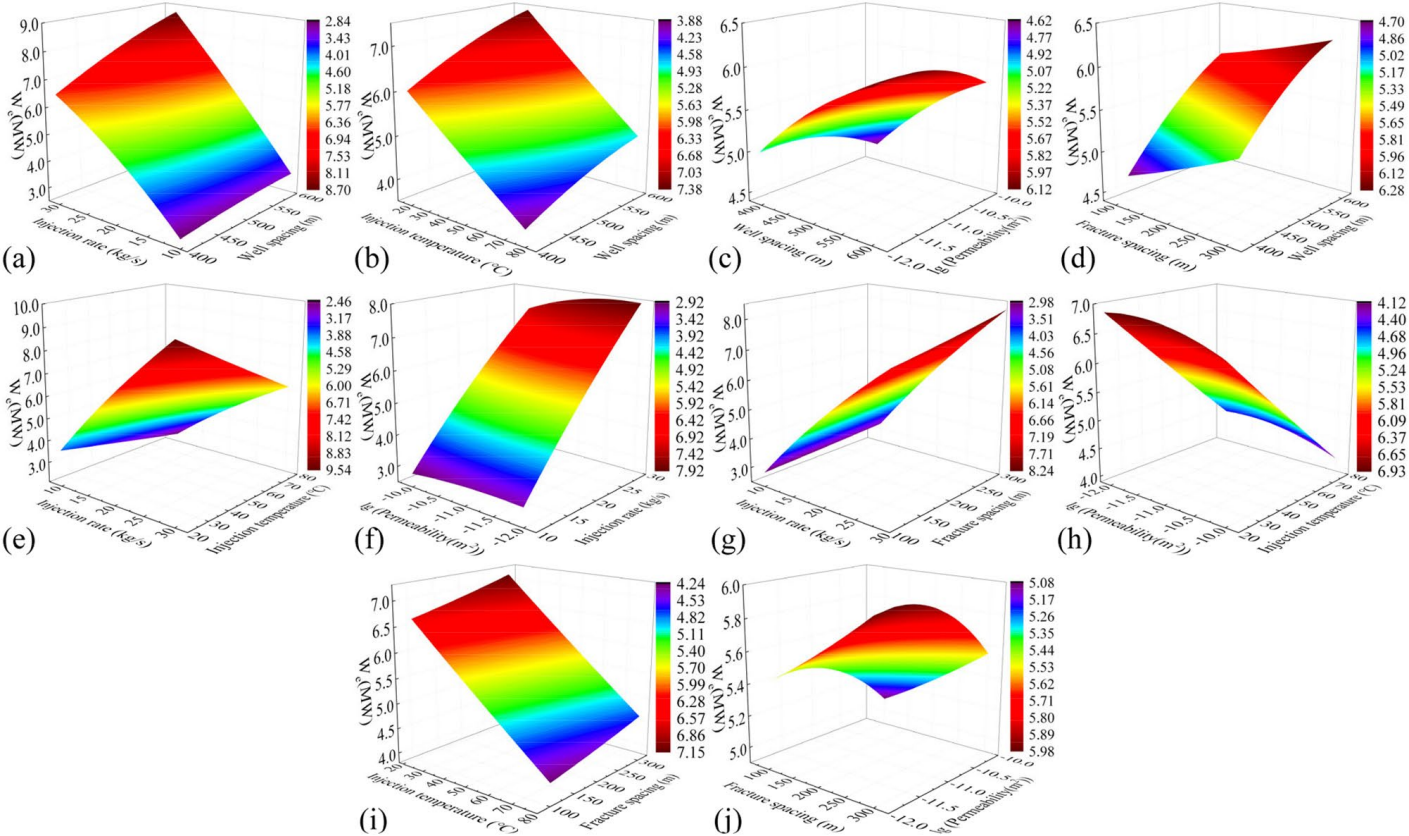
Cloud plot of the electric power $W_{e}$ under the influence of combined factors.

Figure 14 presents cloud plots illustrating the variation of electrical power ($W_{e}$) under the influence of combined factors. The injection rate and injection temperature exhibit nearly linear relationships with $W_{e}$. According to Eq. (3), it can be observed that increasing the injection rate and decreasing the injection temperature directly result in an increase in $W_{e}$. This is because injecting low-temperature fluid into the reservoir at a high speed accelerates temperature reduction and heat recovery in the reservoir. Increasing the injection rate can increase $W_{e}$ by about 5.0 MW, while decreasing the injection temperature can increase $W_{e}$ by about 2.5 MW. Expanding the well spacing enlarges the thermal reservoir volume, while reducing the fracture spacing increases the contact area between the water flow and the reservoir. These modifications collectively improve the heat transfer medium obtaining more heat from the reservoir. Expanding the well spacing and reducing the crack spacing can increase $W_{e}$ by 2.1 MW and 0.83 MW, respectively. Smaller fracture permeability leads to fluid expansion into the thermal storage rock mass surrounding the fracture, and the water flow is sufficiently heated to produce a delayed temperature decline, appropriately reducing the fracture permeability can increase $W_{e}$ by 0.40–0.65 MW. After comparing all the factors, the main influences on $W_{e}$ were ranked as follows: injection rate, injection temperature, well spacing, fracture spacing, and fracture permeability.

#### Thermal efficiency

**Fig. 15:**
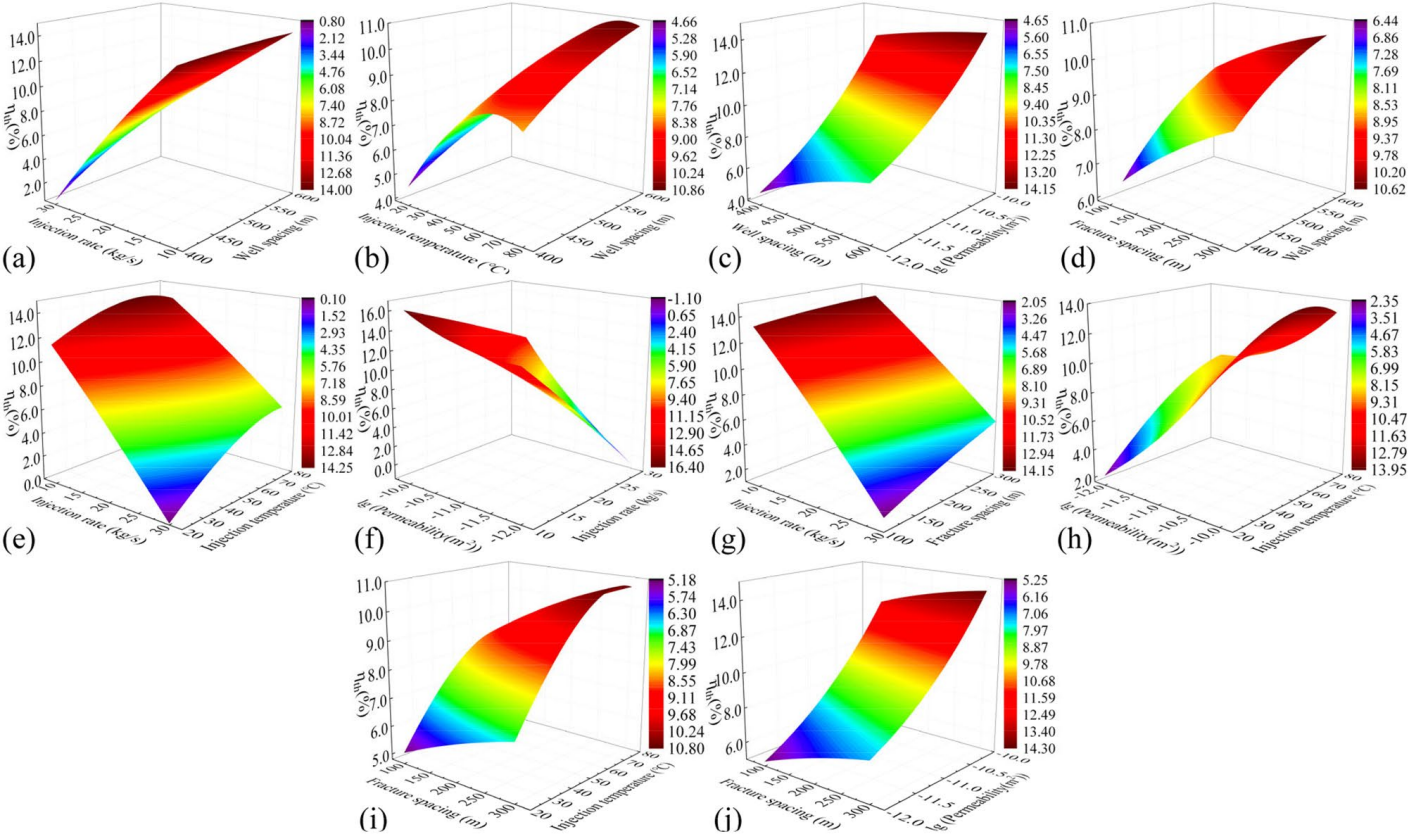
Cloud plot of the thermal efficiency $\eta_{th}$ under the influence of combined factors.

Figure 15 displays cloud plots depicting the variation of thermal efficiency ($\eta_{th}$) under the influence of combined factors. The relationship between injection temperature and $\eta_{th}$ is nonlinear. As the injection temperature increases, the viscosity and resistance of the fluid decrease, resulting in a decrease in internal energy consumption of the system. However, as the injection temperature continues to rise, it leads to a significant decrease in power generation, causing $\eta_{th}$ to first increase and then decrease. Overall, $\eta_{th}$ shows an increase of approximately 3.7–6.3%. The relationship between all other factors and $\eta_{th}$ is roughly linear. Increasing the permeability and decreasing the injection rate boost $\eta_{th}$ by approximately 7.4–11.8% and 11.4–14.3%, respectively. This is mainly due to the significant reduction in internal system losses achieved by these adjustments. Larger well spacing and smaller fracture spacing result in a larger effective heat transfer area, increasing $\eta_{th}$ of the system by around 3% and 2.5%, respectively. However, excessively large well spacing leads to increased internal losses, while excessively small fracture spacing hinders heat transfer in the reservoir, and both scenarios significantly increase the cost of fracturing. After comparing all the factors, the main influences on $\eta_{th}$ were ranked as follows: injection rate, fracture permeability, injection temperature, well spacing, and fracture spacing.

## The optimal strategy for the EGS

The power generation performance of the three horizontal wells EGS in the Zhacang geothermal field is influenced by multiple factors, and it is noteworthy that certain factors exhibit a nonlinear correlation with the performance index of the EGS. These factors are interdependent, resulting in the formation of a complex system. In addition, in the process of optimizing the engineering scheme, it is crucial to recognize the limitations of relying solely on a single evaluation index. This is due to the existence of conflicting objectives among different factors when assessed using diverse indices. For example, while a higher injection rate may improve power generation efficiency, it can also lead to excessive reservoir flow impedance, which contradicts the safety requirements of the engineering project. Therefore, it is necessary to employ scientific and effective methods to assess the significance of each performance indicator of EGS. This will aid in selecting an appropriate thermal development strategy to enhance the power generation capacity of the three horizontal wells in the Zhacang geothermal field.

### The basic principle of AHP

AHP is a straightforward, adaptable, and pragmatic technique for making decisions involving multiple criteria. It was initially introduced during the 1980s by Professor T.L. Saaty, a distinguished American authority in the field of operations research^58^. Its primary advantage lies in its fair and rational categorization system, which allows for flexible synthesis of various evaluation factors. The 9-level proportional scaling method is used, and the corresponding meanings of each scale are shown in Table 4. Drawing from expert experience, the importance of each evaluation indicator is compared pairwise, and the scale is set to generate the judgment matrix of the AHP. By calculating the maximum eigenvalue of the judgment matrix $A$ and its corresponding eigenvectors, normalizing these eigenvectors, the weight coefficients of each assessment index are obtained^59^.

**Table 4:** Scale and meaning.

Scale	Meaning
1	Equally important
3	Slightly important
5	Obviously important
7	Strongly important
9	Extremely important
2,4,6,8	The median of the two adjacent judgments above

(1) Determine the parameter set. The index parameter set for the i-th scheme, $E_{i} = \left\{ {e_{1} ,e_{2} ,e_{3} ,e_{4} ,e_{5} } \right\}$, $i = 1,2,...,n$, representing $T_{50}$, $R$, $I_{R}$, $W_{e}$, $\eta_{th}$.

(2) EGS performance indicators are standardized. Because of the different dimensions of each parameter, it will have an impact on the evaluation. Therefore, the parameters are normalized using the polar deviation normalization method, as shown in Eqs. (13), (14)^56^, and the normalized dimensions are consistent and the correlation between the parameters is unchanged.

The normalized formula for the positive indicator is as follows:

13 $$e_{Nk} = \frac{{e_{k} - \min \left( {e_{k} } \right)}}{{\max \left( {e_{k} } \right) - \min \left( {e_{k} } \right)}},\,k = 1,2,...,5$$

The normalized formula for the negative indicator is as follows:

14 $$e_{Nk} = \frac{{\max \left( {e_{k} } \right) - e_{k} }}{{\max \left( {e_{k} } \right) - \min \left( {e_{k} } \right)}},\,k = 1,2,...,5$$

where $e_{Nk}$ is the normalized value of each indicator, dimensionless; $e_{k}$ is the original value of various performance indicators of EGS; $\max (e_{k} )$ and $\min (e_{k} )$ are represents the maximum and minimum values of each performance indicator.

(3) Based on the importance of the five performance indicators for the evaluation of the power generation capacity of the EGS, a pairwise comparison matrix $A$ was designed as follows:

15 $$A = \left[ {\begin{array}{*{20}c} {} & {T_{50} } & R & {I_{R} } & {W_{e} } & {\eta_{th} } \\ {T_{50} } & 1 & {{1 \mathord{\left/ {\vphantom {1 3}} \right. \kern-0pt} 3}} & {{1 \mathord{\left/ {\vphantom {1 5}} \right. \kern-0pt} 5}} & {{1 \mathord{\left/ {\vphantom {1 6}} \right. \kern-0pt} 6}} & {{1 \mathord{\left/ {\vphantom {1 7}} \right. \kern-0pt} 7}} \\ R & 3 & 1 & {{1 \mathord{\left/ {\vphantom {1 2}} \right. \kern-0pt} 2}} & {{1 \mathord{\left/ {\vphantom {1 4}} \right. \kern-0pt} 4}} & {{1 \mathord{\left/ {\vphantom {1 5}} \right. \kern-0pt} 5}} \\ {I_{R} } & 5 & 2 & 1 & {{1 \mathord{\left/ {\vphantom {1 3}} \right. \kern-0pt} 3}} & {{1 \mathord{\left/ {\vphantom {1 4}} \right. \kern-0pt} 4}} \\ {W_{e} } & 6 & 4 & 3 & 1 & {{1 \mathord{\left/ {\vphantom {1 2}} \right. \kern-0pt} 2}} \\ {\eta_{th} } & 7 & 5 & 4 & 2 & 1 \\ \end{array} } \right]$$

(4) Take the eigenvector of the pairwise comparison matrix A corresponding to the maximum eigenvalue λ as the weight vector:

16 $$w = \left( {w_{1} ,w_{2} ,w_{3} ,w_{4} ,w_{5} } \right)^{T} = \left( {0.0395,0.0855,0.1383,0.2938,0.4428} \right)^{T}$$

(5) In order to ensure good consistency of comparison matrix A, this paper uses the random Consistency Ratio (CR) to determine the consistency of the matrix. After calculation, $CR = 0.0296$, $CR < 0.1$, indicating that the paired comparison matrix of the five indicators meets the consistency requirements, so the resulting weight vector is valid.

(6) The evaluation value of the i-th scheme $U_{i}$ is the product of its standardized indicator parameter set $E_{Ni}$ and weight vector $w$, and by comparing the evaluation values, the advantages and disadvantages of each scheme can be quantitatively described.

17 $$U_{i} = E_{Ni} \times w,\,i = 1,2,...,n$$

### Results of AHP optimization

**Fig. 16:**
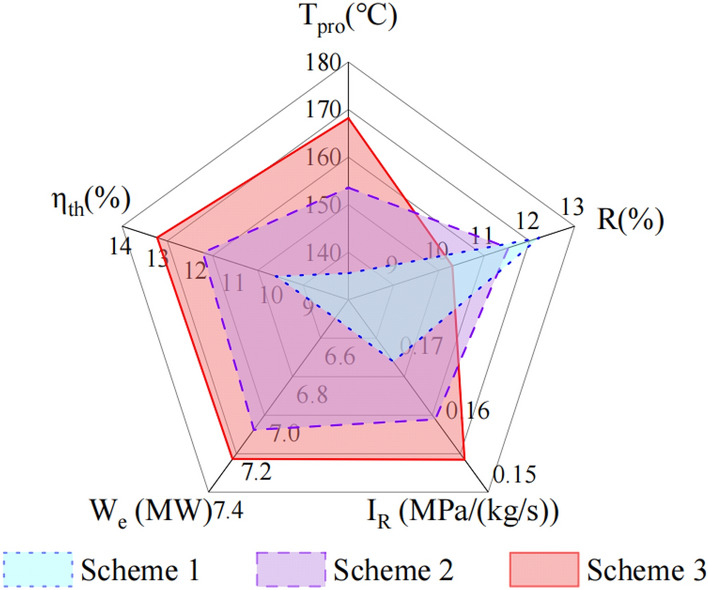
Comparison of the five prediction performance indicators of scheme 1, 2 and 3.

In this study, a comprehensive evaluation system was developed by AHP to assess the power generation performance of three horizontal wells EGS. The weight ordering of the five indicators was calculated by MATLAB: $\eta_{th}$ > $W_{e}$ > $I_{R}$ > $R$ > $T_{50}$. Through the utilization of this evaluation system, millions of schemes are assessed, leading to the identification of the optimal extraction schemes at well spacings of 400 m, 500 m, and 600 m. These schemes are displayed in Table 5, achieving scores of 0.708, 0.754, and 0.770, respectively, which are higher than the baseline model score of 0.545. As illustrated in Fig. 16, scheme 3 outperforms the other two schemes in four indexes, with high heat production, low flow impedance, and large thermal efficiency of the system.

**Table 5:** Comprehensive evaluation results of scheme 1, 2 and 3.

Scheme	d (m)	q (kg/s)	t (℃)	k_f_ (m^2^)	d_f_ (m)	$T_{50}$	$R$	$I_{R}$	$W_{e}$	$\eta_{th}$	Score
1	400	30	50	1 × 10^–10^	100	0.161	0.562	0.849	0.563	0.838	0.708
2	500	29	54	1 × 10^–10^	100	0.399	0.515	0.855	0.629	0.884	0.754
3	600	27	58	1 × 10^–10^	100	0.574	0.429	0.860	0.634	0.916	0.770

**Fig. 17:**
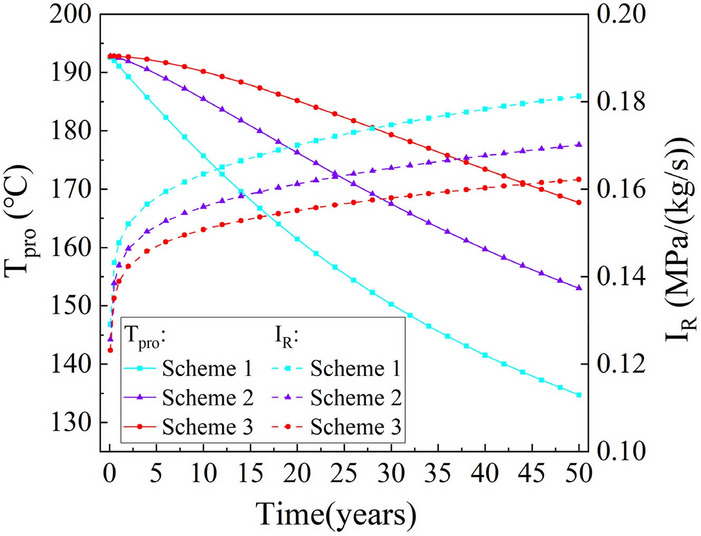
The evolution of the production temperature T_pro_ and flow impedance I_R_ during the 50 year operation time.

The production temperature and flow impedance during 50 years of operation are shown in Fig. 17. It is evident that the production temperature gradually decreases over time. In scheme 1, with a well spacing of 400 m, the production temperature decreased from 192.7 °C to 134.7 °C, representing a total decrease of 30%. In scheme 2, the temperature decreased from 192.7 °C to 153.0 °C over the entire operating period, resulting in an approximate 21% decrease. Scheme 3, with a well spacing of 600 m, has the slowest decline in production temperature, with only a 13% decrease from 192.7 °C to 167.7 °C over 50 years. Garnish suggests that an economically successful EGS plant should have a decline in production temperature of less than 10% over the operating period^46^, which shows that scheme 3 has a very long operating life. During system operation, the flow impedance increases gradually with time, and the average flow impedance for schemes 1, 2, and 3 are 0.17, 0.16, and 0.15 MPa/(kg/s), respectively. These values are all below 0.2 MPa/(kg/s), meeting the commercial requirements.

**Fig. 18:**
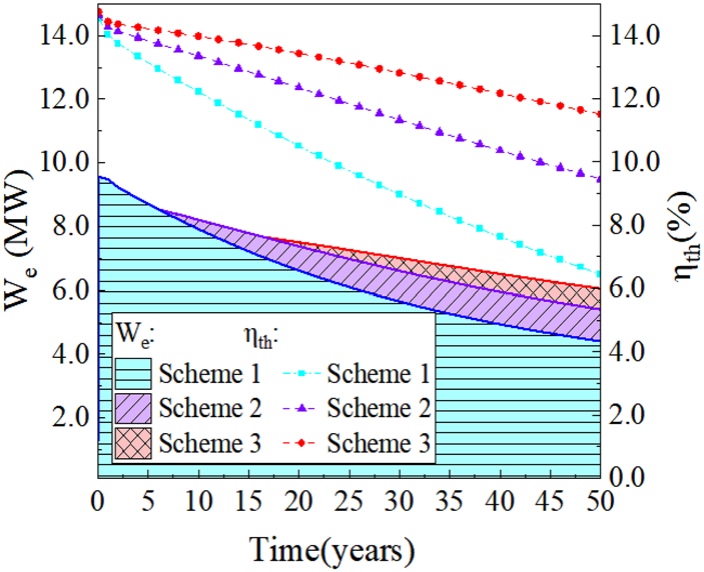
The evolution of the electric power W_e_ and thermal efficiency η_th_ during the 50-year operation time.

The electric power and thermal efficiency change curves are shown in Fig. 18, where the electric power decreases with time during the 50 years of operation. In scheme 1, the electric power decreases from 9.56 MW to 4.41 MW, representing a decrease of approximately 54%. Scheme 2 experiences a decrease from 8.9 MW to 5.41 MW, about 39% reduction in electric power. Similarly, scheme 3 witnesses a decline from 8.17 MW to 6.05 MW, indicating a decrease of around 26%. It is suggested that the decline in electric power should be limited to less than 15% over a 20-year operational period. Scheme 3 manages to achieve a 15% decrease in electric power in the 32nd year, demonstrating compliance with the commercial requirements for EGS power generation performance. By integrating Eq. 3, the total power generation of the system throughout the 50-year operational period can be calculated. The results indicate that scheme 1 achieves a total power generation of 2813.98 GWh, scheme 2 reaches 3095.33 GWh, and scheme 3 obtains 3163.16 GWh. Additionally, the thermal efficiency of scheme 1 exhibits a greater decrease over time, declining from 14.5 to 6.4% over the 50-year period. Scheme 2 experiences a decrease from 14.6 to 9.5%, while scheme 3 demonstrates a smaller reduction, from 14.7 to 11.5%. It can be seen that Scheme 3 is the optimal operation scheme for the three horizontal wells EGS in Zhacang geothermal field.

### Economic efficiency and environmental influence

The major costs of an EGS project include exploration costs, drilling costs, reservoir stimulation costs, equipment installation costs, and Operation And Maintenance (O&M) costs. The estimated investment for geothermal exploration in the Zhacang geothermal field amounts to $4.3 million. The drilling costs comprise vertical well drilling expenses and horizontal well drilling expenses. For instance, the drilling cost of the 5100 m deep GPK4 well completed by Soultz EGS in France in 2004 was $5.14 million. This study assumes a vertical well drilling cost of $1,000/m and a horizontal well drilling cost of $1,500/m, which results in a drilling cost of $5.65 million per well, for a total of $17.0 million for the three wells. Assuming the thermal reservoir of the Zhacang geothermal field is modified by gel proppant fracturing, based on the experience of the oil and gas industry in Daqing Oilfield, the cost of one fracturing treatment is about $0.486 million, and the project will require a total of nine fracturing sessions, totaling $4.4 million. Equipment installation costs heavily depend on the installed capacity, which can be represented by the maximum electrical power output of 8.17 MW. Using an empirical formula, the cost of equipment installation is calculated to be $16.1 million. O&M costs encompass internal energy consumption and other miscellaneous expenses, typically decreasing as the installed capacity increases, which can be calculated based on an empirical formula of $62.9 million.

**Fig. 19:**
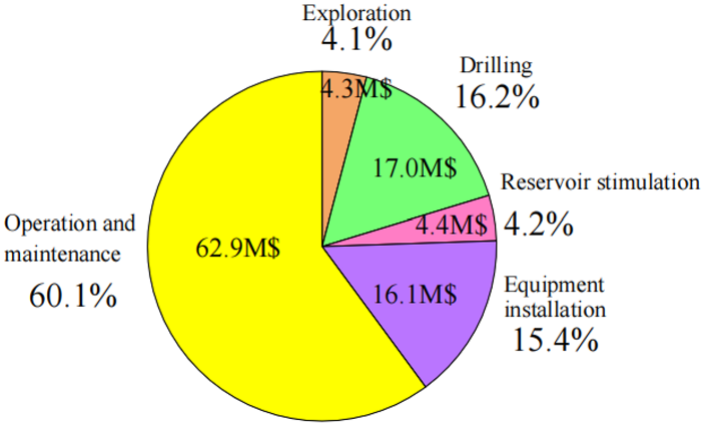
The proportions of costs for different consumption parts.

Based on the above analysis, the projected total expenditure for the proposed EGS power plant is estimated to reach $104.7 million. Figure 19 shows the proportion of costs for the different components. Among these, O&M costs account for about 60% of the total cost, followed by investment in surface equipment and drilling costs at 16% and 15%, respectively. The levelized cost of electricity can be calculated by dividing the total cost by the total amount of electricity generated over 50 years of production (3.163 × 10^6^ MWh). The levelized cost of electricity is estimated at $0.033/kWh, which is lower than the industrial electricity price in Qinghai Province ($0.080/kWh). Consequently, the project demonstrates significant economic benefits, with estimated direct revenue from power generation amounting to as much as $253.1 million.

The indirect benefits of the EGS project are calculated in comparison to conventional fossil fuel power plants. The average GreenHouse Gas (GHG) emissions from geothermal power plants are about 122 g/kWh^60^. In contrast, the International Atomic Energy Agency (IAEA) estimates that electricity generation from fossil fuels emits greenhouse gases equivalent to 460 to 1290 g/kWh. Thus, by replacing a conventional fossil fuel power plant with a geothermal power plant, GHG emissions can be reduced by 338 to 1168 g/kWh. Based on the EGS power plant proposed in this work, the results show that the total savings in GHG emissions over 50 years range from 1.069 to 3.695 Mt. According to the China Carbon Emissions Trading Network, the price of CO_2_ is set at $7.55 per ton, which could result in an indirect income of $27.9 million.

**Fig. 20:**
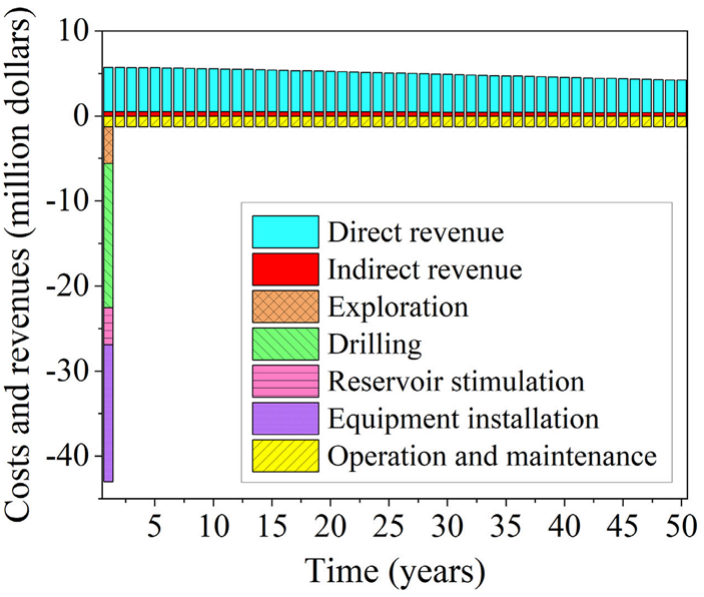
Annual revenue and expenditure histogram for the proposed power plant.

Figure 20 shows a histogram of the annual revenues and expenses of the proposed power plant, with expense concentrated in the first year, mainly for construction costs such as drilling, reservoir stimulation, and equipment installation, and after one year the expenses are mainly for operation and maintenance costs. Revenues are primarily power plant generation and carbon trading revenues. In the first eight years, the expenses of the EGS power plant exceeded its revenues, and from the ninth year onwards, the plant began to generate revenues that increased each year, with cumulative revenues amounting to $172.2 million in the fiftieth year. An analysis of the economic benefits and CO_2_ reductions shows that the proposed EGS power plant is favorable for future development. However, the development of EGS will have environmental impacts, including groundwater pollution and induced seismic activities. During the development and operation of EGS, real-time environmental monitoring should be conducted and a rapid environmental impact assessment mechanism should be established.

## Conclusion

The study of geological and engineering data of Zhacang geothermal field shows that the temperature of granodiorite reservoir at 4200-4400 m depth is over 180 °C, and the direction of the maximum principal stress is NE-SW. The natural fractures in the thermal reservoir are developed, which has the development conditions of high-quality hot dry rock. In this study, the PSO-GA-BPNN intelligent prediction model based on hydrothermal coupling was innovatively constructed, and the optimal development scheme was determined by combining the analytic hierarchy process. The main conclusions are as follows:

1. Comparing the production temperature performance of different models, the PSO-GA-BPNN model has a mean absolute error of 0.196, a mean absolute percentage error of 0.111, and a root mean square error of 0.289 on the test set, indicating that the PSO-GA-BPNN model has high prediction accuracy.
2. The main influencing factors of the three horizontal wells enhanced geothermal system (EGS) are well distance, injection rate, injection temperature, fracture permeability, and fracture spacing. The degree of influence of each factor on the EGS performance index is different, with the injection rate having the most influence and the interaction of fracture permeability with other parameters being the most obvious.
3. The Analytic Hierarchy Process (AHP) is used to evaluate the five indicators of power generation performance of the three horizontal wells, with a weight order of $$\eta_{th}$$ > $$W_{e}$$ > $$I_{R}$$ > $$R$$ > $$T_{50}$$.
4. Based on the evaluation system of AHP, the optimal EGS configuration is determined with a well spacing of 600 m, injection rate of 27 kg/s, temperature of 58° C, fracture permeability of 1 × 10^–10^ m^2^, and spacing of 100 m.
5. The total power generation of the optimal EGS system of three horizontal wells in Zhacang geothermal field is 3163.16 GWh, with a leveling cost of $0.033/kWh. Compared with traditional thermal power, it can reduce greenhouse gases by 1.069–3.695 Mt, with significant economic and environmental benefits.

This method shows good application effect and promotion potential in EGS, which can provide reference for EGS design of three horizontal wells in other geothermal fields. Future research should focus on extending the PSO-GA-BPNN model to heterogeneous or fractured reservoirs and optimizing multi-well EGS configurations (e.g., vertical or radial wells). In addition, other geothermal fields need to be studied to verify the universality of the method and promote the in-depth development of EGS optimization design.

## Data Availability

The data presented in this study are available at https://github.com/hun728/souce-data.

## Abbreviations

BPNN: Back-propagation neural network
PSO: Particle swarm optimization
GA: Genetic algorithm
EGS: Enhanced geothermal system
THM: Thermal–hydraulic–mechanical
GHG: Greenhouse gas
O&M: Operation and maintenance
TCS: Thermal conductivity scanner
BBR: Bending beam rheometer
MINC: Multiple interacting continua
MAE: Mean absolute error
MAPE: Mean absolute percentage
RMSE: Root mean squared error
HDR: Hot dry rock
ANN: Artificial neural network
AHP: Analytic hierarchy process
CR: Consistency ratio
IAEA: International atomic energy agency
*P*: Initial reservoir pressure
*T*_*50*_: The 50th year production temperature
*R*: Heat extraction efficiency
*I*_*R*_: Flow impedance
*W*_*e*_: Electric power
*T*_*pro*_: Production temperature
*W*_*h*_*(t)*: The heat extraction efficiency of the system at time t
*V*: Volume of reservoir
*T*_*r*_: Temperature of the rock reservoir
*Tinj*: Injection temperature
n: Rock porosity
*c*_*r*_: Specific heat capacity of the rock reservoir
*c*_*ω*_: Specific heat capacity of the fluid
*q*: Output flow rate
*h*_*pro*_: Specific enthalpy of effluent
*h*_*inj*_: Specific enthalpy of water injection
*P*_*inj*_: Injection pressure
*P*_*pro*_: Bottom-hole pressure of the production well
*f*: Proportion of total heat production
*T*_*rej*_: Heat rejection temperature
*W*_*p*_: Average internal energy consumption of the EGS
*W*_*h*_: Average heat recovery rate of EGS
*g*: Acceleration of gravity
*h*_*1*_: Depth of the injection well
*h*_*2*_: Depth of the production well
*P*_*best*_: The best position of the individual
*g*_*best*_: The best global position
*x*: Particle position
*i*: The i-th particle
*j*: Dimension
*k*: Current number
*P*_*i*_: Individual optimal position
*P*_*g*_: Group optimal solution of particle
$$c_{1}$$: Learning factors
$$c_{2}$$: Learning factors
$$r_{1}$$: Random values
$$r_{2}$$: Random values
*X*^***^: Standardized data samples
*X*: Data samples
*X*_*max*_: Maximum values of data samples
*X*_*min*_: Minimum values of data samples
*y*: Actual value
*y*^*'*^: Predicted value
*R*^*2*^: Coefficient of determination
*A*: Judgment matrix
*e*_*Nk*_: Normalized value of each indicator
*e*_*k*_: The original value of various performance indicators of EGS
max(*e*_*k*_): Maximum values of each performance indicator
min(*e*_*k*_): Minimum values of each performance indicator
*U*_*i*_: The evaluation value of the i-th scheme
*E*_*Ni*_: Product of standardized indicator parameter set
*w*: Weight vector
$$\eta_{th}$$: Thermal efficiency
$\Gamma_{50}$: Total operating time
$\rho_{r}$: Reservoir rock density
$$\rho_{w}$$: Fluid density
$$\rho$$: Water density
$\eta_{p}$: Efficiency of the pump
$$v$$: Particle velocity
$$\omega$$: Inertia weight
λ: Maximum eigenvalue
A: Pairwise comparison matrix
***: Standardization
*'*: Predicted
*50*: The 50th
*R*: Impedance
*e*: Electric
*th*: Thermal
*pro*: Production
*h*: Heat
50: 50 Years
*r*: Rock
*inj*: Injection
*ω*: Fluid
*rej*: Rejection
*p*: Internal energy & pump
*1*: Injection well
*2*: Production well
*i*: Individual & the i-th
*g*: Group
*max*: Maximum values
*min*: Minimum values
*Nk*: Normalized
*k*: Original
*Ni*: Standardized indicator
